# Supplementary material for: Protecting brains and saving futures guidelines: A prospective, multicenter, and observational study on the use of telemedicine for neonatal neurocritical care in Brazil
Source: PLoS One. 2022 Jan 12;17(1):e0262581. doi: 10.1371/journal.pone.0262581 (PMC8754327; doi:10.1371/journal.pone.0262581)
Supplement: S2 Appendix — (PDF) [file pone.0262581.s002.pdf]

S2 Appendix. Characteristics of Participants Centers

1. Irmandade da Santa Casa de Misericórdia de São Paulo – SP: Philanthropic hospital
2. Hospital e Maternidade Santa Joana – SP: Private hospital
3. Maternidade Pró Matre Paulista – SP: Private hospital
4. Hospital e Maternidade Santa Maria – SP: Private hospital
5. Perinatal - Unidade Barra – RJ: Private hospital
6. Perinatal - Unidade Laranjeiras – RJ: Private hospital
7. Perinatal - Unidade Icaraí – RJ: Private hospital
8. Perinatal - Unidade Teresópolis – RJ: Private hospital
9. Hospital Estivadores de Santos – SP: Public hospital
10. UTI Neonatal Nicola Albano - Unidade Campos de Goytacazes – RJ: Private hospital
11. UTI Neonatal Nicola Albano - Unidade Macaé – RJ: Private hospital
12. Hospital Sepaco – SP: Private hospital
13. Hospital Regional Jorge Hosmann – SP: Public hospital
14. Hospital Santa Luzia – DF: Private hospital
15. Hospital São Cristóvão – SP: Private hospital
16. Hospital Unimed - Unidade Volta Redonda – RJ: Private hospital
17. Hospital Santa Lúcia – DF: Private hospital
18. Hospital Santa Helena – DF: Private hospital
19. Fundação Santa Casa de Misericórdia do Pará – PA: Public foundation
20. Hospital da Criança e Maternidade – SP: Philanthropic foundation
