# Supplementary material for: Protecting brains and saving futures guidelines: A prospective, multicenter, and observational study on the use of telemedicine for neonatal neurocritical care in Brazil
Source: PLoS One. 2022 Jan 12;17(1):e0262581. doi: 10.1371/journal.pone.0262581 (PMC8754327; doi:10.1371/journal.pone.0262581)
Supplement: S3 Appendix — (PDF) [file pone.0262581.s003.pdf]

S3 Appendix - The Protecting Brains and Saving Futures (PBSF) Guidelines

1. **Equipment and Resources**: each center received the necessary brain monitoring equipment, protocols, procedures and service logistics.

1.1 Equipment

a) *Video aEEG / EEG:* Continuous, real-time, non-invasive video & brain electrical activity monitoring. The device used is the Neuron-Spectrum-3 system (Neurosoft, Ivanono, Russia), with the software module for aEEG and trending.

b) *NIRS:* Hemodynamic evaluation through the application of sensors to infer cerebral/somatic tissue oxygenation and blood flow. The utilized device was INVOS^{TM} 5100C Cerebral/Somatic Oximeter (Meditronic, Minneapolis, MN, USA).

c) *Whole-Body cooling device*: Equipment necessary for precise temperature control in infants with moderate or severe HIE receiving therapeutic hypothermia. A target temperature value of 33.5 °C is maintained for 72 hours followed by slow rewarming (from 0.2 to 0.5^{o}C/hour). Different hypothermia equipment is utilized across the centers including Blanketrol III (Cincinatti Sub-Zero), HICO-Aquatherm 660, Medi-Therm II (Gaymar), and Artic Sun (BD). One center uses ice-gel packs.

1.2. Clinical protocols

As part of PBSF services, each participating center receives clinical protocols that remain easily accessible for consultation by the medical and nursing staff.

a) *Neuro-NICU*: includes description of neonatal neurocritical care including brain monitoring methodologies (video aEEG/EEG and NIRS) with guidance on installation of equipments, indications for monitoring, and results interpretation. (Appendix I)

b) *Therapeutic Hypothermia (TH)*: criteria for indication of TH and recommendations for clinical management during cooling (Appendix II).

c) *Neonatal Seizures*: includes description of main causes of neonatal seizures, clinical and electroencephalographic diagnosis, and available therapies (Appendix III).

1.3. Procedures

After installation and training with the necessary equipment and clinical protocols, PBSF promotes connexion between the associated NICU and the remote monitoring center called the *Central of Surveillance and Intelligence (CSI)*. Thus, brain monitoring data from aEEG/EEG and NIRS, and vital signs acquired from multiparametric equipment at the bedside, are sent to CSI using a standard HL7 or proprietary protocol, depending on the availability of the manufacturers of each equipment at the bedside. This information is uploaded through the internet to a cloud system and can be remotely assessed by the PBSF staff for evaluation, report and live feedback. The following data security precautions and protocols have been developed:

a) All local devices have a security system that provides malware protection, and its management and update are controlled by a central cloud.

b) All locations have equipment data backup capabilities with advanced technology deduplication, encryption and compression, optimizing data transmission bandwidth and ensuring an additional layer of security against ransomware.

c) In the CSI, there are monitoring capabilities of all connected equipment ensuring the integrity of all devices and high availability.

d) Communication between the local equipment and the central server is done with encrypted data, protecting the privacy of transmitted data.

e) Access to the monitoring system, management of backup data, and security services are provided through authentication mechanisms for users, so that only those duly registered with identification and password can access patient information or data stored in the cloud.

1.4. Service Logistics

After training the local staff and providing connection with CSI, the bedside team of each center becomes responsible to indicate brain monitoring and notify the CSI team. Electrodes and sensors placement are done by trained nurses and internet connection is checked. The remote team at CSI is comprise of neonatologists, nurse specialist, and a neurologist / neurophysiologist who assists reviewing cases of suspected seizures (for final diagnosis), participates in management decisions, provides support in challenging cases and interpretation of the aEEG/EEG exams. In addition, the neurologist also reads full video EEGs performed in selected cases and perform consultations through telemedicine in cases where the local medical team request specialized assistance for the diagnosis and management of critically ill infants.

The CSI team is available 24 hours a day all year around, allowing not only case discussions but also generating simplified reports of brain monitoring information on the patient monitor's display at each site, every 06 hours. If seizures are detected by the remote team, the bedside clinician is contacted by phone call and a message is also displayed at the patient

monitor. According to the monitoring findings, interventions such as use of anticonvulsants, change in ventilation parameters or blood transfusion may be suggested by remote medical staff. Finally, at the end of each monitoring period, the remote medical staff provides a written report of the brain monitoring findings. All data is stored in a protected cloud database.

2. **Training**: Training and teaching of all health care professional team of each NICU are central steps. Details of each training session are provided below.

2.1. Initial workshop

The initial training conducted with every center that starts participation with PBSF consists of educational sessions conducted through a workshop with the objective of introducing the concept of a Neonatal Neurological ICU or Brain Focused NICU care, and familiarization with specifics patient care protocols, technology and equipment. Furthermore, clinical cases are discussed to illustrate routine practice. The workshop is individualized according to the characteristics and demands of each center, but at least nine introductory classes, one of them hands-on with 3 stations, are carried out as detailed below:

a. Principles, recordings and patterns of continuous brain monitoring with aEEG.
b. Use of aEEG in clinical practice at the Neonatal ICU.
c. Perinatal asphyxia and therapeutic hypothermia: principles and impact of treatment.
d. Therapeutic hypothermia protocol: indication and management.
e. Use of aEEG in neonatal seizures.
f. NIRS: introduction, rationale and principles.
g. NIRS: case studies.

h. Hands-on sessions: **Station 1** - Clinical cases of HIE: indication of therapeutic hypothermia; **Station 2** - Video aEEG / EEG and **Station 3** - NIRS: learning how to use the equipment.

i. Steps for interpreting the aEEG and interactive Quiz.

2.2. Long-term educational plan

PBSF continuing education consists of monthly videoconferences and discussion of clinical cases from each center, chosen by the NICU medical team. Furthermore, some sessions are held to present collected data and results to each center, allowing discussions for improvements and better patient care. Each center is responsible for encouraging their local health professionals to participate in these videoconferences. They are presented by invited speakers or members of the PBSF team. The subjects proposed for videoconferences are dynamic. As an example, for the year of 2019 the following topics were presented/discussed:

a. Neurological Neonatal ICU and implementation of an advanced telemedicine model.
b. Principles of aEEG: installation and interpretation.
c. HIE and therapeutic hypothermia: eligible patient criteria and clinical management.
d. Use of aEEG for neonatal seizures diagnosis and treatment.
e. aEEG Monitoring in HIE, prematurity and other useful clinical scenarios.
f. aEEG clinical case discussion.
g. NIRS: introduction and principles.
h. NIRS clinical case discussion.
i. Use of cranial ultrasound and brain MRI in the NICU: indications, classifications and results

j. Neonatal Neurodevelopmental Care

k. Mild HIE

l. Review on HIE / Hypothermia

m. Follow-up of high-risk newborns for brain injury

3. **Recordings of biological signals**: Each patient monitoring is multiparametric, customized and include:

a) Video aEEG / EEG. The electroencephalographic monitoring is performed continuously by using two aEEG / EEG channels and video imaging and accessed remotely by experienced users. The following findings are analyzed: a) background activity: continuous normal voltage, discontinuous, continuous low voltage, burst suppression and isoelectric (flat); b) sleep-wake cycling: developed, immature or absent; c) seizures: absent, isolated, repetitive or status epilepticus. The number of subclinical seizures will also be evaluated.

b) Pulse oximetry (SpO_2). Pulse oximetry determines oxygen saturation by measuring the absorption of red and infrared light passed through the tissues. Changes in absorption caused by pulsation of blood in the vascular bed are used to determine oxygen saturation and pulse rate. Oxygen saturation percent is calculated with each pulse detected, and thus the monitor display is continually updated.

c) Non-invasive blood pressure (NIBP). After proper blood pressure cuff placement, measurement is done by oscillometry with reducing deflation method. At the completion of a measurement cycle the systolic and diastolic pressures are displayed. Blood pressure measurements may be initiated manually, or automatically at time intervals determined by the user.

d) Heart rate (HR). The pulse rate, in beats per minute, will be determined primarily from the SpO2 measurement methodology and displayed continuously.

e) Near Infrared Spectroscopy. Neonatal sensors are used for simultaneous cerebral and somatic saturation monitoring. Near-infrared light emitted from a light source passes through the infant's underlying skin and tissue and is partially absorbed by oxygenated and deoxygenated hemoglobin before being reflected back to a detector on the same sensor. A tissue saturation level (rSO2) is then calculated and continuously displayed in the monitor reflecting a ratio of arterial and venous blood (25%:75%) and the balance between oxygen delivery and consumption in the underlying tissue. Signals are sent to the central server, through a standard Health Level 7 or proprietary protocol (depending on each equipment at the bedside).

4. Clinical and Imaging Data

Clinical and imaging data is collected and includes demographic (sex, maternal age, race), clinical (birth weight, gestational age, Apgar scores, diagnosis of early-onset sepsis, presence of clinical seizure, need for ventilatory support, surfactants, and vasoactive drugs, need for surgeries, length of hospital stay and mortality) and imaging data including cranial ultrasound (cUS) and brain MRI findings.
