# Supplementary material for: Protecting brains and saving futures guidelines: A prospective, multicenter, and observational study on the use of telemedicine for neonatal neurocritical care in Brazil
Source: PLoS One. 2022 Jan 12;17(1):e0262581. doi: 10.1371/journal.pone.0262581 (PMC8754327; doi:10.1371/journal.pone.0262581)
Supplement: S4 Appendix — (PDF) [file pone.0262581.s004.pdf]

S4. Appendix - Minimal anonymized data set.

Patient #	
Center #	
Year	
Indications for PBSF Guideline	
Gestational Age (weeks)	
Apgar 5	
Apgar 10	
Adherence*	
Primary Outcome 1	
Primary Outcome 2	
Primary Outcome 3	
Primary Outcome 4	
Primary Outcome 5	
Primary Outcome 6	
Clinical*	
Primary Outcome 1	
Primary Outcome 2	
Primary Outcome 3	
Primary Outcome 4	
Primary Outcome 5	
Primary Outcome 6	

*Data all primary outcomes specified in the protocol will be made available.
