# Supplementary material for: Protecting brains and saving futures guidelines: A prospective, multicenter, and observational study on the use of telemedicine for neonatal neurocritical care in Brazil
Source: PLoS One. 2022 Jan 12;17(1):e0262581. doi: 10.1371/journal.pone.0262581 (PMC8754327; doi:10.1371/journal.pone.0262581)
Supplement: S1 File — (PDF) [file pone.0262581.s005.PDF]

PARECER CONSUBSTANCIADO DO CEP

DADOS DO PROJETO DE PESQUISA

Título da Pesquisa: Protecting Brains and Saving Futures: estudo observacional de um protocolo de neuroproteção por telemedicina em unidades de terapia intensiva neonatal

Pesquisador: Gabriel Fernando Todeschi Variane

Área Temática:

Versão: 1

CAAE: 04526818.2.1001.5479

Instituição Proponente: IRMANDADE DA SANTA CASA DE MISERICORDIA DE SAO PAULO

Patrocinador Principal: Financiamento Próprio

DADOS DO PARECER

Número do Parecer: 3.142.318

Apresentação do Projeto:

Introdução: A lesão cerebral perinatal é comumente presente em recém-nascidos prematuros, em consequência de hemorragia intracraniana, infarto
cerebral focal e lesão cerebral hipóxica isquêmica secundária à hipóxia-isquêmica intraparto. Ambas as lesões são identificadas no momento dos
sintomas clínicos, isto é, convulsões ou apnéia, o que limita o potencial para a prevenção. A monitorização com o eletroencefalograma de amplitude
integrada, Espectroscopia de Infravermelho Próximo e a intervenção com a hipotermia terapêutica é uma ferramenta promissora na unidade de
terapia intensiva neuro-neonatal. Apesar do benefício descrito, estima-se que menos de 5% dos centros neonatais brasileiros utilizem hipotermia
terapêutica ou monitoramento cerebral contínuo para o tratamento de recém-nascidos de alto risco. A fim de reduzir a lacuna existente, o modelo
avançado de telemedicina proposto poderia ser uma alternativa à proteção neuro nos países em desenvolvimento. Método: Trata-se de um estudo
prospectivo multicêntrico de coorte observacional realizado em 20 unidades de terapia intensiva neonatal no Brasil. O período de recrutamento será
de 5 anos. Os pacientes serão avaliados após a alta hospitalar entre 18 e 24 meses de vida. Foram incluídos todos os bebês internados em uma das

unidades de terapia intensiva neonatal que foram indicados para realizar a monitorização cerebral com dois canais de aEEG com os seguintes
critérios de inclusão: encefalopatia hipóxico-isquêmica, prematuridade extrema, hemorragia peri-intraventricular grave, congênita doença cardíaca,
malformações cerebrais, infecções congênitas, sepse tardia, erros inatos do metabolismo, período pós-cardiorrespiratório e também convulsões por
várias causas. Discussão: O estudo poderá avaliar a viabilidade de estabelecer um modelo de telemedicina para fornecer assistência remota a
neonatos com alto risco de lesão cerebral, adesão ao protocolo, pacientes submetidos à hipotermia terapêutica. Além disso, os achados das
imagens, os dados de morbimortalidade e neurodesenvolvimento serão correlacionados.

Objetivo da Pesquisa:

Objetivo Primário:

avaliar a aplicabilidade e a eficácia de um modelo de assistência neonatal com o auxílio da telemedicina (Protocolo PBSF).

Objetivo Secundário:

verificar o efeito de achados de monitorização cerebral contínua (incluindo aEEG/EEG e NIRS) com achados de morbimortalidade e alterações no
neurodesenvolvimento em RN de alto risco.

Avaliação dos Riscos e Benefícios:

Riscos:

perda de confidencialidade que é minimizado pois os dados são protegidos por criptografia.

Benefícios:

atenção especializada à distância para centros que, em tese, não teriam esse recurso no serviço local.

Comentários e Considerações sobre a Pesquisa:

pesquisa pertinente, com forte evidencia cientifica, formato adequado

Considerações sobre os Termos de apresentação obrigatória:

apresenta todos documentos obrigatorios adequados

Conclusões ou Pendências e Lista de Inadequações:

aprovado

Considerações Finais a critério do CEP:

Este parecer foi elaborado baseado nos documentos abaixo relacionados:

Tipo Documento	Arquivo	Postagem	Autor	Situação
Declaração de Instituição e Infraestrutura	Of_ACPC_2672018.pdf	13/12/2018 08:06:27	Patrícia Sant Ana	Aceito
Informações Básicas do Projeto	PB_INFORMAÇÕES_BÁSICAS_DO_PROJETO_1241144.pdf	07/12/2018 10:53:39		Aceito
Declaração de Instituição e Infraestrutura	Autoriza.pdf	07/12/2018 10:42:59	Gabriel Fernando Todeschi Variane	Aceito
TCLE / Termos de Assentimento / Justificativa de Ausência	TCLE.pdf	07/12/2018 10:42:40	Gabriel Fernando Todeschi Variane	Aceito
Declaração de Pesquisadores	Compromisso.pdf	07/12/2018 10:18:10	Gabriel Fernando Todeschi Variane	Aceito
Orçamento	Form_orcamemto.pdf	06/12/2018 19:35:53	Gabriel Fernando Todeschi Variane	Aceito
Cronograma	Form_crono.pdf	06/12/2018 19:35:00	Gabriel Fernando Todeschi Variane	Aceito
Projeto Detalhado / Brochura Investigador	PBSF_15_12_18.pdf	06/12/2018 19:08:25	Gabriel Fernando Todeschi Variane	Aceito
Parecer Anterior	parecer_cientifica.pdf	05/12/2018 15:27:35	Gabriel Fernando Todeschi Variane	Aceito
Folha de Rosto	Folha_rosto_assinada.pdf	05/12/2018 13:59:45	Gabriel Fernando Todeschi Variane	Aceito

Situação do Parecer:

Aprovado

Necessita Apreciação da CONEP:

Não

Endereço: SANTA ISABEL
Bairro: VILA BUARQUE **CEP:** 01.221-010
UF: SP **Município:** SAO PAULO
Telefone: (11)2176-7689 **Fax:** (11)2176-7688 **E-mail:** cepsc@santacasasp.org.br

SAO PAULO, 12 de Fevereiro de 2019

Assinado por:
José Cassio de Moraes
(Coordenador(a))

Endereço: SANTA ISABEL
Bairro: VILA BUARQUE **CEP:** 01.221-010
UF: SP **Município:** SAO PAULO
Telefone: (11)2176-7689 **Fax:** (11)2176-7688 **E-mail:** cepsc@santacasasp.org.br
