# Supplementary material for: Protecting brains and saving futures guidelines: A prospective, multicenter, and observational study on the use of telemedicine for neonatal neurocritical care in Brazil
Source: PLoS One. 2022 Jan 12;17(1):e0262581. doi: 10.1371/journal.pone.0262581 (PMC8754327; doi:10.1371/journal.pone.0262581)
Supplement: S2 File — (PDF) [file pone.0262581.s006.PDF]

AYRTON MUGNAINI JR.
Tradutor público e intérprete comercial - Inglês/Português
Endereço: R. França Carvalho, 372 - 03183-070 - São Paulo - SP - Brasil
Telefone: 0 xx 11 2768-1766 / e-mail: millediscchi@yahoo.com.br
Registrado na Junta Comercial do Estado de São Paulo sob o no. 661
RG: 8.728.873-4 (SSP) / CPF: 890.985.448-00 / CCM: 2.936.528-7

TRADUÇÃO No. 958 – LIVRO 13 – PÁGINA 3456

On the 15th of December, 2020, I received one OPINION ON CLINICAL RESEARCH, written in Portuguese, and which I translate into English as follows:

====

SANTA CASA DE MISERICÓRDIA DE SÃO PAULO
[SÃO PAULO CHARITY HOSPITAL]

CONSUBSTANCED OPINION OF THE RESEARCH ETHICS COMMITTEE

RESEARCH PROJECT DATA
Research Title: Protecting Brains and Saving Futures: observational study of a neuroprotection protocol by telemedicine in neonatal intensive care units
Researcher: Gabriel Fernando Todeschi Variane
Thematic Area: [blank]
Version: 1
CAAE: 04526818.2.1001.5479
Proposing Institution: THE BROTHERHOOD OF THE SANTA CASA DE MISERICÓRDIA DE SÃO PAULO
Main Sponsor: Self-Financing

OPINION DATA
Opinion Number: 3,142,318

Introduction: Perinatal brain injury is commonly present in premature newborns, as a result of intracranial haemorrhage, focal cerebral infarction and hypoxic ischemic brain injury secondary to intrapartum hypoxia-ischemic. Both lesions are identified at the time of clinical symptoms, that is, seizures or apnea, which limits the potential for prevention. Monitoring with the Amplitude Intgrated Electroencephalography, Near Infrared Spectroscopy and intervention with therapeutic hypothermia is a promising tool in the neuro-neonatal intensive care unit. Despite the benefit described, it is estimated that less than 5% of Brazilian neonatal centers use therapeutic hypothermia or continuous brain monitoring for the treatment of high-risk newborns. In order to reduce the existing gap, the proposed advanced telemedicine model could be an alternative to neuro protection in developing countries. Method: This is a study prospective multicenter observational cohort performed in 20 neonatal intensive care units in Brazil. The recruitment period will be 5 years. Patients will be evaluated after hospital discharge between 18 and 24 months of life. All babies hospitalized in one of the neonatal intensive care units that were indicated to perform brain monitoring with two aEEG channels have been included, with the following inclusion criteria: hypoxic-ischemic encephalopathy, extreme prematurity, severe peri-intraventricular haemorrhage, congenital heart disease, brain malformations, congenital infections, late sepsis, innate errors of metabolism, post-cardiorespiratory period and also seizures from various causes.

Discussion: The study may assess the feasibility of establishing a telemedicine model to provide remote assistance to neonates at high risk of brain injury, adherence to the protocol, patients undergoing therapeutic hypothermia. In addition, the findings of the images, data on morbidity and mortality and neurodevelopment will be correlated.

Research Objective:
Primary Objective:
To evaluate the applicability and effectiveness of a neonatal care model with the aid of telemedicine (PBSF Protocol).
Secondary Objective:
To verify the effect of continuous brain monitoring findings (including an EEG/EEG and NIRS) with morbidity and mortality findings and changes in neurodevelopment in high-risk newborns.

Assessment of Risks and Benefits:
Risks:
Loss of confidentiality which is minimized as the data is protected by encryption.
Benefits:
Specialized remote care for centres that, in theory, would not have this resource in the local service.

Ayrton Mugnaini Jr.
Tradutor Público
Intérprete Comercial
Registro JUCESP nº 661

AYRTON MUGNAINI JR.
Tradutor público e intérprete comercial - Inglês/Português
Endereço: R. França Carvalho, 372 - 03183-070 - São Paulo - SP - Brasil
Telefone: 0 xx 11 2768-1766 / e-mail: milledischi@yahoo.com.br
Registrado na Junta Comercial do Estado de São Paulo sob o no. 661
RG: 8.728.873-4 (SSP) / CPF: 890.985.448-00 / CCM: 2.936.528-7

Research comments and considerations:
Pertinent research, with strong scientific evidence, appropriate format

Mandatory submission terms considerations:
It presents all appropriate mandatory documents

Conclusions or Issues and List of Inadequacies:
Approved

Final Considerations at the discretion of the Research Ethics Committee:
This opinion was prepared based on the documents listed below:

Type of Document	File	Date of Posting	Author	Status
Statement of Institution and Infrastructure	Of_ACPC_2672018.pdf	13thDec2018, 08:06:27	Patrícia Sant Ana	Accepted
Basic Project Information	PB_INFORMAÇÕES_BÁSICAS_DO_PROJETO_1241144.pdf	7th Dec 2018, 10:53:39		Accepted
Statement of Institution and Infrastructure	Autoriza.pdf	7thDec2018, 10:42:59	Gabriel Fernando Todeschi Variane	Accepted
IC/Terms of Assent/ Institution and Infrastructure Statement	Autoriza.pdf	7thDec2018, 10:42:59	Gabriel Fernando Todeschi Variane	Accepted
IC/Terms of Assent/ Justification of Absence	TCLE.pdf	7thDec2018, 10:42:40	Gabriel Fernando Todeschi Variane	Accepted
Declaration from Researchers	Compromisso.pdf	7thDec2018, 10:18:10	Gabriel Fernando Todeschi Variane	Accepted
Budget	Form_orcamemto.pdf	6thDec2018, 19:35:53	Gabriel Fernando Todeschi Variane	Accepted
Schedule	Form_crono.pdf	6thDec2018, 19:35:00	Gabriel Fernando Todeschi Variane	Accepted
Detailed project/ Brochure Investigator	PBSF_15_12_18.pdf	6thDec2018, 19:08:25	Gabriel Fernando Todeschi Variane	Accepted
Previous Opinion	parecer_cientifica.pdf	5thDec2018, 15:27:35	Gabriel Fernando Todeschi Variane	Accepted
Front Page	Folha_rosto_assinada.pdf	5thDec2018, 13:59:45	Gabriel Fernando Todeschi Variane	Accepted

Situation of Opinion:
Approved

Need for the National Commission on Ethics in Research Approval:
No

São Paulo, the 12th of February 2019.

Signed by:
José Cassio de Moraes
(coordinator)

M-

Ayrton Mugnaini Jr.
Tradutor Público
Intérprete Comercial
Registro JUCESP nº 661

AYRTON MUGNAINI JR.
Tradutor público e intérprete comercial - Inglês/Português
Endereço: R. França Carvalho, 372 - 03183-070 - São Paulo - SP - Brasil
Telefone: 0 xx 11 2768-1766 / e-mail: milledischi@yahoo.com.br
Registrado na Junta Comercial do Estado de São Paulo sob o no. 661
RG: 8.728.873-4 (SSP) / CPF: 890.985.448-00 / CCM: 2.936.528-7

====

This is a faithful English translation of the document that was presented to me, to which I bear witness.

São Paulo, the 15th of December, 2020.

Ayrton Mugnaini Jr.
Sworn Translator

Ayrton Mugnaini Jr.
Tradutor Público
Intérprete Comercial
Registro JUCESP nº 661
