# Supplementary material for: Protecting brains and saving futures guidelines: A prospective, multicenter, and observational study on the use of telemedicine for neonatal neurocritical care in Brazil
Source: PLoS One. 2022 Jan 12;17(1):e0262581. doi: 10.1371/journal.pone.0262581 (PMC8754327; doi:10.1371/journal.pone.0262581)
Supplement: S3 File — (PDF) [file pone.0262581.s007.PDF]

PARECER CONSUBSTANCIADO DO CEP

DADOS DA EMENDA

Título da Pesquisa: Protecting Brains and Saving Futures: Estudo observacional de um protocolo de neuroproteção por telemedicina em Unidades de Terapia Intensiva Neonatal

Pesquisador: Gabriel Fernando Todeschi Variane

Área Temática:

Versão: 2

CAAE: 04526818.2.1001.5479

Instituição Proponente: IRMANDADE DA SANTA CASA DE MISERICORDIA DE SAO PAULO

Patrocinador Principal: Financiamento Próprio

DADOS DO PARECER

Número do Parecer: 3.357.239

Apresentação do Projeto:

Projeto aprovado por este CEP em fevereiro de 2019, porém autores anexaram emenda na plataforma brasil

Objetivo da Pesquisa:

Objetivo Primário:

avaliar a aplicabilidade e a eficácia de um modelo de assistência neonatal com o auxílio da telemedicina (Protocolo PBSF).

Objetivo Secundário:

verificar o efeito de achados de monitorização cerebral contínua (incluindo aEEG/EEG e NIRS) com achados de morbimortalidade e alterações no
neurodesenvolvimento em RN de alto risco.

Avaliação dos Riscos e Benefícios:

Riscos:

perda de confidencialidade que é minimizado pois os dados são protegidos por criptografia.

Benefícios:

atenção especializada à distância para centros que, em tese, não teriam esse recurso no serviço local.

Comentários e Considerações sobre a Pesquisa:

Autores não destacaram o objetivo da nova emenda e nem se ocorreram mudanças no projeto original, já aprovado por este CEP

Considerações sobre os Termos de apresentação obrigatória:

adequados, aprovados anteriormente

Recomendações:

explicar o motivo da emenda e destacar alterações realizadas no projeto

Conclusões ou Pendências e Lista de Inadequações:

ressaltar alterações no projeto incluídas na plataforma brasil

Considerações Finais a critério do CEP:

Este parecer foi elaborado baseado nos documentos abaixo relacionados:

Tipo Documento	Arquivo	Postagem	Autor	Situação
Informações Básicas do Projeto	PB_INFORMAÇÕES_BÁSICAS_1323049_E1.pdf	26/04/2019 08:08:28		Aceito
Declaração de Instituição e Infraestrutura	Of_ACPC_2672018.pdf	13/12/2018 08:06:27	Patrícia Sant Ana	Aceito
Declaração de Instituição e Infraestrutura	Autoriza.pdf	07/12/2018 10:42:59	Gabriel Fernando Todeschi Variane	Aceito
TCLE / Termos de Assentimento / Justificativa de Ausência	TCLE.pdf	07/12/2018 10:42:40	Gabriel Fernando Todeschi Variane	Aceito
Declaração de Pesquisadores	Compromisso.pdf	07/12/2018 10:18:10	Gabriel Fernando Todeschi Variane	Aceito
Orçamento	Form_orcamemto.pdf	06/12/2018 19:35:53	Gabriel Fernando Todeschi Variane	Aceito
Cronograma	Form_crono.pdf	06/12/2018 19:35:00	Gabriel Fernando Todeschi Variane	Aceito
Projeto Detalhado / Brochura Investigador	PBSF_15_12_18.pdf	06/12/2018 19:08:25	Gabriel Fernando Todeschi Variane	Aceito
Parecer Anterior	parecer_cientifica.pdf	05/12/2018 15:27:35	Gabriel Fernando Todeschi Variane	Aceito
Folha de Rosto	Folha_rosto_assinada.pdf	05/12/2018 13:59:45	Gabriel Fernando Todeschi Variane	Aceito

Situação do Parecer:
Aprovado

Necessita Apreciação da CONEP:
Não

SAO PAULO, 30 de Maio de 2019

Assinado por:
Paulo Augusto Ayroza Galvão Ribeiro
(Coordenador(a))

Endereço: Rua Marques de Itu, 381
Bairro: VILA BUARQUE **CEP:** 01.223-001
UF: SP **Município:** SAO PAULO
Telefone: (11)2176-1818 **Fax:** (11)2176-7688 **E-mail:** cepsc@santacasasp.org.br
