# Supplementary material for: Protecting brains and saving futures guidelines: A prospective, multicenter, and observational study on the use of telemedicine for neonatal neurocritical care in Brazil
Source: PLoS One. 2022 Jan 12;17(1):e0262581. doi: 10.1371/journal.pone.0262581 (PMC8754327; doi:10.1371/journal.pone.0262581)
Supplement: S4 File — (PDF) [file pone.0262581.s008.PDF]

AYRTON MUGNAINI JR.
Tradutor público e intérprete comercial - Inglês/Português
Endereço: R. França Carvalho, 372 - 03183-070 - São Paulo - SP - Brasil
Telefone: 0 xx 11 2768-1766 / e-mail: milledischi@yahoo.com.br
Registrado na Junta Comercial do Estado de São Paulo sob o no. 661
RG: 8.728.873-4 (SSP) / CPF: 890.985.448-00 / CCM: 2.936.528-7

TRADUÇÃO No. 959 – LIVRO 13 – PÁGINA 3459

On the 15th of December, 2020, I received one OPINION ON CLINICAL RESEARCH, written in Portuguese, and which I translate into English as follows:

====

SANTA CASA DE MISERICÓRDIA DE SÃO PAULO
[SÃO PAULO CHARITY HOSPITAL]

CONSUBSTANCED OPINION OF THE RESEARCH ETHICS COMMITTEE

AMENDMENT DATA
Research Title: Protecting Brains and Saving Futures: observational study of a neuroprotection protocol by telemedicine in neonatal intensive care units
Researcher: Gabriel Fernando Todeschi Variane
Thematic Area: [blank]
Version: 2
CAAE: 04526818.2.1001.5479
Proposing Institution: THE BROTHERHOOD OF THE SANTA CASA DE MISERICÓRDIA DE SÃO PAULO
Main Sponsor: Self-Financing

OPINION DATA
Opinion Number: 3,357,239

Project Presentation:
Project approved by this Research Ethics Committee in February 2019, but the authors have annexed an amendment at the Brazilian platform.

Research Objective:
Primary Objective:
To evaluate the applicability and effectiveness of a neonatal care model with the aid of telemedicine (PBSF Protocol).
Secondary Objective:
To verify the effect of continuous brain monitoring findings (including aEEG/EEG and NIRS) with morbidity and mortality findings and changes in neurodevelopment in high-risk newborns.

Assessment of Risks and Benefits:
Risks:
Loss of confidentiality which is minimized as the data is protected by encryption.
Benefits:
Specialized remote care for centres that, in theory, would not have this resource in the local service.

Research Comments and Considerations:
The authors have not stated the objective of the new amendment nor if there have been changes in the original project, already approved by this Research Ethics Committee.

Mandatory submission terms considerations:
Adequate, previously approved

Recommendations:
Explain the reason for the amendment and state changes made in the project

Conclusions or issues and list of inadequacies:
State changes in the project included in the brasil platform

M.

Ayrton Mugnaini Jr.
Tradutor Público
Intérprete Comercial
Registro JUCESP nº 661

AYRTON MUGNAINI JR.
Tradutor público e intérprete comercial - Inglês/Português
Endereço: R. França Carvalho, 372 - 03183-070 - São Paulo - SP - Brasil
Telefone: 0 xx 11 2768-1766 / e-mail: milledischi@yahoo.com.br
Registrado na Junta Comercial do Estado de São Paulo sob o no. 661
RG: 8.728.873-4 (SSP) / CPF: 890.985.448-00 / CCM: 2.936.528-7

Final Considerations at the discretion of the Research Ethics Committee:
This opinion was prepared based on the documents listed below:

Type of Document	File	Date of Posting	Author	Status
Basic Project Information	PB_INFORMAÇÕES_BÁSICAS_1323049_E1.pdf	26th Apr 2018, 08:08:28		Accepted
Statement of Institution and Infrastructure	Of_ACPC_2672018.pdf	13thDec2018, 08:06:27	Patrícia Sant Ana	Accepted
Statement of Institution and Infrastructure	Autoriza.pdf	7thDec2018, 10:42:59	Gabriel Fernando Todeschi Variane	Accepted
IC/Terms of Assent/ Justification of Absence	TCLE.pdf	7thDec2018, 10:42:40	Gabriel Fernando Todeschi Variane	Accepted
Declaration from Researchers	Compromisso.pdf	7thDec2018, 10:18:10	Gabriel Fernando Todeschi Variane	Accepted
Budget	Form_orcamemto.pdf	6thDec2018, 19:35:53	Gabriel Fernando Todeschi Variane	Accepted
Schedule	Form_crono.pdf	6thDec2018, 19:35:00	Gabriel Fernando Todeschi Variane	Accepted
Detailed project/ Brochure Investigator	PBSF_15_12_18.pdf	6thDec2018, 19:08:25	Gabriel Fernando Todeschi Variane	Accepted
Previous Opinion	parecer_cientifica.pdf	5thDec2018, 15:27:35	Gabriel Fernando Todeschi Variane	Accepted
Front Page	Folha_rosto_assinada.pdf	5thDec2018, 13:59:45	Gabriel Fernando Todeschi Variane	Accepted

Situation of Opinion:
Approved

Need for the National Commission on Ethics in Research Approval:
No

São Paulo, the 30th of May 2019.

Signed by:
Paulo Augusto Ayroza Galvão Ribeiro
(coordinator)

====

This is a faithful English translation of the document that was presented to me, to which I bear witness.

São Paulo, the 15th of December, 2020.

Ayrton Mugnaini Jr.
Sworn Translator

Ayrton Mugnaini Jr.
Tradutor Público
Intérprete Comercial
Registro JUCESP nº 661
