# Supplementary material for: Protecting brains and saving futures guidelines: A prospective, multicenter, and observational study on the use of telemedicine for neonatal neurocritical care in Brazil
Source: PLoS One. 2022 Jan 12;17(1):e0262581. doi: 10.1371/journal.pone.0262581 (PMC8754327; doi:10.1371/journal.pone.0262581)
Supplement: S6 File — (PDF) [file pone.0262581.s010.PDF]

AYRTON MUGNAINI JR.
Tradutor público e intérprete comercial - Inglês/Português
Endereço: R. França Carvalho, 372 - 03183-070 - São Paulo - SP - Brasil
Telefone: 0 xx 11 2768-1766 / e-mail: milledischi@yahoo.com.br
Registrado na Junta Comercial do Estado de São Paulo sob o no. 661
RG: 8.728.873-4 (SSP) / CPF: 890.985.448-00 / CCM: 2.936.528-7

TRADUÇÃO No. 960 – LIVRO 13 – PÁGINA 3461

On the 15th of December, 2020, I received one OPINION ON CLINICAL RESEARCH, written in Portuguese, and which I translate into English as follows:

====

SANTA CASA DE MISERICÓRDIA DE SÃO PAULO
[SÃO PAULO CHARITY HOSPITAL]

CONSUBSTANCED OPINION OF THE RESEARCH ETHICS COMMITTEE

AMENDMENT DATA
Research Title: Protecting Brains and Saving Futures: observational study of a neuroprotection protocol by telemedicine in neonatal intensive care units
Researcher: Gabriel Fernando Todeschi Variane
Thematic Area: [blank]
Version: 3
CAAE: 04526818.2.1001.5479
Proposing Institution: THE BROTHERHOOD OF THE SANTA CASA DE MISERICÓRDIA DE SÃO PAULO
Main Sponsor: Self-Financing

OPINION DATA
Opinion Number: 3,506,106

Project Presentation:
Amendment done for inclusion of the participating centres.

Research Objective:
Not applicable

Assessment of Risks and Benefits:
Not applicable

Research Comments and Considerations:
Changes done continues as a higilight in the process

Mandatory submission terms considerations:
Inclusion of 15 participating centres

Conclusions or issues and list of inadequacies
No issues

M.

Ayrton Mugnaini Jr.
Tradutor Público
Intérprete Comercial
Registro JUCESP nº 661

AYRTON MUGNAINI JR.

Tradutor público e intérprete comercial - Inglês/Português

Endereço: R. França Carvalho, 372 - 03183-070 - São Paulo - SP - Brasil
Telefone: 0 xx 11 2768-1766 / e-mail: milledischi@yahoo.com.br
Registrado na Junta Comercial do Estado de São Paulo sob o no. 661
RG: 8.728.873-4 (SSP) / CPF: 890.985.448-00 / CCM: 2.936.528-7

Final Considerations at the discretion of the Research Ethics Committee:
This opinion was prepared based on the documents listed below:

Type of Document	File	Date of Posting	Author	Status
Basic Project Information	PB_INFORMAÇÕES_BÁSICAS_1372324_E2.pdf	4th Jun 2019, 15:45:09		Accepted
Detailed project/ Brochure Investigator	PBSF_Projeto.pdf	4th Jun 2019, 15:45:42	Gabriel Fernando Todeschi Variane	Accepted
Statement of Institution and Infrastructure	Of_ACPC_2672018.pdf	13thDec2018, 08:06:27	Gabriel Fernando Todeschi Variane	Accepted
Statement of Institution and Infrastructure	Autoriza.pdf	7thDec2018, 10:42:59	Patrícia Sant Ana	Accepted
IC/Terms of Assent/ Justification of Absence	TCLE.pdf	7thDec2018, 10:42:40	Gabriel Fernando Todeschi Variane	Accepted
Declaration from Researchers	Compromisso.pdf	7thDec2018, 10:18:10	Gabriel Fernando Todeschi Variane	Accepted
Budget	Form_orcamemto.pdf	6thDec2018, 19:35:53	Gabriel Fernando Todeschi Variane	Accepted
Schedule	Form_crono.pdf	6thDec2018, 19:35:00	Gabriel Fernando Todeschi Variane	Accepted
Previous Opinion	parecer_cientifica.pdf	5thDec2018, 15:27:35	Gabriel Fernando Todeschi Variane	Accepted
Front Page	Folha_rosto_assinada.pdf	5thDec2018, 13:59:45	Gabriel Fernando Todeschi Variane	Accepted

Situation of Opinion:
Approved

Need for the National Commission on Ethics in Research Approval:
No

São Paulo, the 13th of August 2019.

Signed by:
Pollyanna Oliveira Lira
(coordinator)

====

This is a faithful English translation of the document that was presented to me, to which I bear witness.

São Paulo, the 15th of December, 2020.

Ayrton Mugnaini Jr.
Sworn Translator

Ayrton Mugnaini Jr.
Tradutor Público
Intérprete Comercial
Registro JUCESP nº 661
