# Supplementary material for: Protecting brains and saving futures guidelines: A prospective, multicenter, and observational study on the use of telemedicine for neonatal neurocritical care in Brazil
Source: PLoS One. 2022 Jan 12;17(1):e0262581. doi: 10.1371/journal.pone.0262581 (PMC8754327; doi:10.1371/journal.pone.0262581)
Supplement: S7 File — (PDF) [file pone.0262581.s011.PDF]

*P*rotecting *B*rains and *S*aving *F*utures: Estudo observacional de um protocolo de neuroproteção por telemedicina em unidades de terapia intensiva neonatal

Descritores (Keywords)

Recém-nascido, telemedicina, eletroencefalografia de amplitude integrada, monitoração do cérebro.

Introdução (Introduction)

A neonatologia é uma das especialidades médicas que mais evoluíram nas últimas décadas. O incremento do surfactante, óxido nítrico, ventilação protetora promoveram verdadeira revolução na redução da mortalidade neonatal. Entretanto, por diversas vezes esse incremento na sobrevida não foi acompanhado de um neurodesenvolvimento adequado. Múltiplas são as afecções no período neonatal que estão associadas a alto risco de desenvolvimento de sequelas. O primeiro grupo de risco que merece destaque inclui os recém nascidos com asfixia perinatal, doença com incidência de um a oito por 1.000 nascidos vivos a termo[1] e que representa a terceira causa mais comum de morte neonatal (23%), após nascimento prematuro (28%) e infecções graves (26%)[2-4]. Apesar do importante avanço citado no cuidado perinatal nas últimas décadas, a asfixia continua a ser uma condição grave, levando à denominada encefalopatia hipóxico-isquêmica (EHI).

O recém-nascido com encefalopatia grave apresenta um risco muito alto de morte, paralisia cerebral e retardo mental. Aqueles com encefalopatia moderada podem apresentar déficits motores significativos, deficiência motora fina, comprometimento da memória, disfunção visual, hiperatividade e atraso na prontidão escolar[5-8]. Outra condição que merece fundamental destaque é a prematuridade, sobretudo pelo risco de de déficits neurológicos. Estudos epidemiológicos[8,9] apontam que no mundo nascem 1,15 milhões de asfixiados e 13 milhões de prematuros ao ano. Destes bebês, 233.000 asfixiados e 350.000 prematuros irão evoluir com sequelas neurológicas moderadas/graves. Outras disfunções também envolvem riscos importantes de lesão neurológica permanente, destacando-se: cardiopatia congênita, malformações cerebrais, infecções congênitas, sepse tardia, hemorragia peri intraventricular, erros inatos do metabolismo, período pós parada-cardiorrespiratória e crises convulsivas por causas diversas[6-8].

Sendo assim, torna-se cada vez mais comum e relevante a implantação do modelo de unidade de terapia intensiva neonatal (UTIN) neurológica, ambiente altamente especializado,

com foco em prover estratégias para prevenção de sequelas neurológicas na população de risco, destacando-se aqui, hipotermia terapêutica e monitoramento cerebral contínuo. Ensaios clínicos randomizados foram conduzidos para avaliar terapias neuroprotetoras, em especial a hipotermia terapêutica (HT), direcionada aos pacientes com encefalopatia hipóxico-isquêmica (EHI). O resfriamento provou diminuir morte e minimizar deficiências incapacitantes em lactentes aos 18-24 meses de idade[10-15]. Adicionalmente, meta-análises recentes relatam que a hipotermia é eficaz e segura[16,17]. Experiência bem-sucedida na implantação de hipotermia terapêutica também já foi descrita em centros brasileiros[18].

A abordagem protetora da lesão cerebral em bebês com EHI transcende a HT. A asfixia perinatal representa a principal causa de crises convulsivas no período neonatal, representando de 40 a 60% da etiologia de crises em bebês a termo[19-22]. No entanto, mais de 80% das crises epilépticas[23,24] e até mesmo estados de mal epiléptico, dentro da UTIN, são completamente subclínicos. Portanto, o manejo apropriado do paciente com asfixia carece do monitoramento da atividade elétrica cerebral de base e do reconhecimento de crises convulsivas. Para isso o uso do Eletroencefalograma de Amplitude Integrada (aEEG), associado à leitura de EEG bruto mostrou-se metodologia segura e útil.

Alterações visualizadas de forma dinâmica na atividade de base e atividade epiléptica estão relacionadas a função e injúria cerebral. A interpretação em tempo real permite ações muito mais rápidas e assertivas em relação ao quadro clínico vigente[25-27]. Em prematuros e asfixiados a presença de crises epilépticas é fator isolado de risco para atrasos no neurodesenvolvimento[28-31]. O reconhecimento e o tratamento imediato reduzem a sua duração e tem relação com melhor neurodesenvolvimento[32].

Outro recurso para monitoramento cerebral é a medida não invasiva da perfusão regional, mais precisamente, a cerebral e a renal. A perfusão inadequada ou flutuante, indicativa de auto regulação ineficiente, também pode resultar em injúria cerebral. A Espectroscopia Infravermelha Proximal ou comumente conhecida como *Near Infrared Spectroscopy* (NIRS), permite a triagem e a avaliação neurológica à beira-leito por método não invasivo por longos períodos de tempo e sem efeitos adversos[33-35].

O Brasil conta com cerca de três milhões de nascidos vivos ao ano e estima-se que destes, parcela significativa de bebês tenham alto risco para injúria cerebral[36].

Como qualquer país continental em desenvolvimento, os recursos disponíveis para cuidados neonatais são extremamente desiguais e variam de unidades terciárias neonatais de localização mais central e bem estruturadas, a outras totalmente desprovidas dos mesmos.

A telemedicina pode ser aplicada como ferramenta para educação, consulta,

atendimento e pesquisa. Esse modelo é a evolução natural para a transformação do cuidado de saúde no mundo digital, cujo produto principal é a redução da distância entre dois locais, que promove acesso e alcance de metodologias específicas, além de reduzir custo de estrutura. Especificamente na área da saúde, telemedicina define-se como o intercâmbio do uso da informação médica de um lugar a outro, via comunicação eletrônica, para melhorar a situação da saúde do paciente[37-39].

No que se refere à educação, pode ser entregue mediante links audiovisuais interativos ao vivo, transmissão de vídeo ao vivo ou visualização de material educativo armazenado.

As tele consultas por sua vez, podem existir da forma tradicional, cujo paciente de outra localização visualiza e ouve as orientações do médico em outro ponto, no mesmo momento, ou de modo alternativo, a exemplo de cardiologistas que emitem laudo a distância, não em tempo real, de imagens ecocardiográficas armazenadas[38].

Na mesma linha conceitual, a tele pesquisa abrange a translação da pesquisa originária em centros acadêmicos aos médicos de assistência primária, na condução de estudos e pode ampliar a abrangência no recrutamento e seguimento de pacientes, análise de resultados provenientes de amostras colaborativas entre centros de pesquisa de todo o mundo, o que favorece o processo de disseminação da ciência e do conhecimento[39].

O desenvolvimento tecnológico ainda promete disrupção e inclusão de outros formatos, o que provavelmente demandará discussões e regulações adicionais[39].

Embora tais cuidados inovadores atraiam os consumidores de serviços de saúde porque podem ser mais rápidos, mais convenientes e mais acessíveis do que um atendimento convencional, o efeito sobre a segurança do paciente e a aplicabilidade em determinados ambientes da prática assistencial merecem atenção especial.

Considerando que a implementação de HT e monitoramento cerebral contínuo pressupõe serviço técnico contínuo e qualificado e recursos e equipamentos especializados, e ainda estimando que menos de 5% dos centros neonatais brasileiros pratiquem esse atendimento de neuroproteção a recém-nascidos de alto risco de forma estruturada[36], justifica-se o uso do modelo de telemedicina avançada que propõe sustentação à lacuna estabelecida no país. O objetivo é fornecer treinamento longitudinal, implantar protocolos e metodologias, e consequentemente prestar assistência apropriadamente especializada em centros remotos.

Com o intuito de fornecer cuidado neurointensivo neonatal a uma gama mais ampla de pacientes, em detrimento à distância, desigualdade de recursos e capacitação especializada[37,38], elaborou-se um protocolo de telemedicina avançada de monitoramento e proteção neurológica, intitulado *Protecting Brains & Saving Futures* (PBSF). O protocolo consiste na aplicação de

metodologias, que incluem o diagnóstico precoce de disfunções neurológicas e consequente terapia neuroprotetora em RN de alto risco, de forma a reduzir a chance de sequelas neurológicas.

Frente ao exposto, este estudo pretende verificar a exequibilidade do modelo do protocolo PBSF, descrito a seguir, em centros de UTIN de diferentes localidades.

Objetivos (Objctives)

O objetivo primário é avaliar a aplicabilidade e a eficácia de um modelo de assistência neonatal com o auxílio da telemedicina (Protocolo PBSF). De modo secundário inclui-se verificar o efeito de achados de monitorização cerebral contínua (incluindo aEEG/EEG e NIRS) com achados de morbimortalidade e alterações no neurodesenvolvimento em RN de alto risco.

Método (Methods)

Desenho e local do estudo (Study design and participating units) - Trata-se de um estudo multicêntrico de coorte observacional, de temporalidade híbrida, ou seja, parte dos dados será obtida de modo retrospectivo (de julho de 2017 até a aprovação do projeto pelo Comitê de ética em Pesquisa - CEP) e parte de modo prospectivo (período após a aprovação do CEP). O período pretendido de recrutamento dos pacientes será de dez anos. O seguimento de cada paciente está previsto para o período durante a hospitalização. Adicionalmente ocorrerá uma avaliação de acompanhamento pós-alta hospitalar entre 18 e 24 meses de vida da criança.

O estudo será realizado em 20 UTIN de centros em diferentes regiões no Brasil, com o intuito de verificar a aplicabilidade do Protocolo PBSF. Além da instituição proponente (Irmandade da Santa Casa de Misericórdia de São Paulo – SP), serão 19 centros coparticipantes do estudo:

1. UTI Neonatal Nicola Albano – Unidade Campos dos Goytacazes – RJ
2. UTI Neonatal Nicola Albano – Unidade Macaé – RJ
3. Hospital e Maternidade Santa Joana – SP
4. Pro Matre Paulista – SP
5. Hospital e Maternidade Santa Maria – SP
6. Perinatal – Unidade Barra – RJ
7. Perinatal – Unidade Laranjeiras – RJ
8. Perinatal – Unidade Icaraí – RJ
9. Perinatal – Unidade Teresópolis – RJ
10. Hospital dos Estivadores de Santos – SP
11. Hospital SEPACO – SP
12. Hospital Regional Jorge Rossmann – SP
13. Hospital Santa Luzia – DF
14. Hospital São Cristovão – SP
15. Hospital Santa Lúcia – DF

16. Hospital Unimed Volta Redonda – RJ
17. Santa Casa de Misericórdia de Belém – PA
18. Hospital Santa Helena – DF
19. Hospital da Criança e Maternidade – SP

Aspectos éticos (Ethical aspects)- O estudo seguirá os preceitos de boas práticas clínicas e será iniciado após aprovação do Comitê de ética em pesquisa. Adicionalmente o projeto receberá autorização formal da Diretoria Clínica e Administrativa de cada centro. Será oferecida aos responsáveis pelos pacientes a participação na pesquisa e, após explicação pela equipe médica, os responsáveis que concordarem serão convidados a assinar um termo de consentimento livre e esclarecido. Para a porção retrospectiva do estudo sempre que possível será também solicitada o consentimento dos responsáveis. Cabe lembrar que serão apenas coletados dados secundários e que não permitem identificação dos sujeitos de pesquisa.

Declaração de conflito de interesses (conflict of interest) - Declara-se que a PBSF comercializa serviços de monitoramento cerebral remota e de neuroproteção em crianças de até um ano de vida.

Amostra (Sample)

Critério de inclusão (Inclusion criteria) – Todos os pacientes admitidos nas UTIN dos centros, de zero a 365 dias de vida que receberam a indicação de monitoramento cerebral aEEG e/ou NIRS pelo centro, o que inclui condição de alto risco de injúria cerebral, a saber, asfixia perinatal, EHI, prematuridade extrema, cardiopatia congênita, malformações cerebrais, infecções congênitas, sepse tardia, hemorragia peri-intraventricular, erros inatos do metabolismo, período pós parada-cardiorrespiratória ou crises convulsivas por causas diversas.

Critério de exclusão (Exclusion criteria) - pacientes com síndromes genéticas ou malformação incompatível com a vida, ou mais de 365 dias de vida.

Protocolo PBSF (PBSF neurocritical guidelines)

Trata-se de um modelo de telemedicina avançada para implantação de UTI Neonatal Neurológica e adota, de um modo sintético, os seguintes pilares:

Implantação de recursos (Resources implementation): metodologias, tecnologia dura e leve necessárias para estruturar o modelo de UTIN. O objetivo deste fundamento é

promover um ambiente capaz de prestar assistência especializada, segura e refinada ao RN com alto risco de injúria cerebral, de forma a reduzir significativamente a evolução de sequelas neurológicas desta população e melhorar a qualidade de vida futura. Trata-se, portanto, de prover a cada centro equipamentos necessários, protocolos, procedimentos e logística de atendimento. Dentre os equipamentos estão:

Vídeo-aEEG/EEG: monitor cerebral contínuo e em tempo real, de caráter não invasivo, e que permite avaliação neurológica atual e prognóstica por meio da análise de traçado eletroencefalográfico.

NIRS: que permite a avaliação hemodinâmica cerebral, através da aplicação de optodos/sensores no escalpe craniano para inferir a oxigenação e fluxo sanguíneo tecidual. Estudos demonstram que alterações de normalidade podem ter relações prognósticas em RN com asfixia, podem estar associadas a perda do mecanismo de auto regulação cerebral, além de correlações com injúria cerebral como hemorragia intracraniana em prematuros[45,46].

HT: consiste em um tratamento que visa a redução da temperatura corpórea do RN para valor alvo de 33,5°C, durante o período de 72 horas após o nascimento. Seu uso tem papel fundamental na redução da mortalidade, diminuição da gravidade da lesão cerebral e melhora da qualidade de vida do paciente[15-17].

Ensino e treinamento (Teaching and training): inicial e longitudinal, in loco, associado a videoconferências mensais com os centros associados.

No sentido de efetivar o uso dos recursos, considera-se esta etapa de suma importância, sobretudo ao elucidar o raciocínio clínico que embasa a utilização do protocolo PBSF. Para tanto, detalha-se a seguir o conteúdo mínimo compartilhado no início da implantação incluindo reciclagens em 06, 12 e 24 meses.

i. Aula 01: O que é o Modelo UTI Neonatal Neurológica?

ii. Aula 02: Manejo específico do paciente com asfixia perinatal.

iii. Aula 03: Implantação e treinamento de Protocolo de Hipotermia Terapêutica.

iv. Aula 04: Ensino sobre o uso e evidência científica do Monitoramento Cerebral Contínuo Vídeo aEEG/EEG e NIRS.

v. Aula 05: Implantação e treinamento de Monitorização Cerebral.

vi. Aula 06: Provendo conexão e interação entre a UTIN Neurológica e a central

de monitoramento (CVI – Central de Vigilância e Inteligência).

vii. Videoconferências Mensais: Encontros mensais entre os centros, através de videoconferências para discussão de protocolos e casos clínicos no sentido de prover treinamento longitudinal.

Implantação de Central de Monitoramento e Conexão ininterrupta (Implementation of the Monitoring central station with continuous conection): (24 horas contínua) com cada centro permitindo promoção de assistência remota e aplicação de monitoramento cerebral.

Desenvolvimento – Quando inicia uma avaliação e monitoramento de um RN de um centro, os dados do paciente, ou seja, traçados eletroencefalográficos e de espectroscopia são conectados a uma central de monitoramento denominada Central de Vigilância e Inteligência (CVI), em tempo real e posteriormente armazenados em um *storage* na nuvem e protegidos. O Protocolo PBSF promove atendimento de equipe especializada instalada na CVI, que segue diretrizes baseadas em forte evidência científica. A equipe é acessada remota e ininterruptamente como sistema de assistência diagnóstica e terapêutica a todos os centros. O monitoramento é multiparamétrico e customizado para cada paciente, utilizando-se de aEEG/EEG, ECG, saturação de pressão de O2 (SpO2), pressão arterial periférica não invasiva (NIBP), frequência cardíaca (FC), Capnografia (pCO2) e NIRS, o que permite correlações entre os achados para precisão diagnóstica.

O monitoramento eletroencefalográfico é realizado pelo uso dois canais aEEG/EEG associado a vídeo imagem e acessados por avaliadores experientes remotamente onde serão avaliados:

- Atividade elétrica de Base: classificada em padrão contínuo de voltagem normal, descontínuo, contínua de baixa voltagem, supressão e isoelétrico,
- Ciclo sono-vigília: desenvolvido, imaturo ou ausente;
- Crise epiléptica: ausente, isolada, repetitiva ou estado de mal epiléptica.

O fluxo sanguíneo cerebral (FSC) será mensurado pelo NIRS de dois canais (cerebral e somático), em termos de valores de tendência em determinados intervalos de tempo.

Adicionalmente, mediante algoritmos avançados que interpretam os sinais vitais, o sistema notifica situações que necessitam de intervenções e transmite um sinal ao profissional

da CVI quanto ao médico do centro. Dentro de cada centro estão instalados os equipamentos de monitoramento, cujos sinais reproduzidos são enviados para um servidor central, através de um protocolo padrão HL7 ou protocolo proprietário, dependendo da disponibilização dos fabricantes de cada equipamento à beira do leito.

No Servidor Central, um software específico reproduz em tela, os mesmos sinais visualizados à beira do leito, permitindo que o médico especialista monitore o paciente de forma específica (monitoramento cerebral), associada a visão sistêmica do paciente, bem como transmite para a central, a imagem em tempo real da criança de modo contínuo.

Por sua vez, um sistema analítico se encarrega de analisar todos os sinais vitais, buscar correlações entre os mesmos e gerar sinais indicativos de anormalidades com vistas a suportar a tomada de decisão dos médicos plantonistas.

Medidas de desfecho (Outcomes of interest)

Desfecho primário (Primary outcomes) – definido como a aplicabilidade do protocolo PBSF que será mensurada pela produção (output) realizada nessa modalidade, a saber:

- Número de horas de monitoramento remoto de pacientes de alto risco
- Número de laudos emitidos de exames aEEG/EEG com ou sem auxílio do NIRS.
- Número de crises epilépticas (clínicas e subclínicas) identificadas remotamente.
- Número de pacientes que realizaram HT.
- Adesão ao protocolo de UTI Neonatal Neurológica (Anexo I).
- Adesão ao protocolo de inclusão de hipotermia terapêutica (Anexo II).
- Número de comunicações remotas com a equipe dos centros.
- Número de intervenções secundárias decorrentes a achados de monitoramento remoto como: administração de anticonvulsivantes, alterações de parâmetros ventilatórios e hemodinâmicos e transfusão sanguínea.
- Número de reuniões de discussão de casos clínicos.

Desfecho secundário (secondary outcomes) -

- Associação de achados de patológicos de monitoramento cerebral (eletrográfico com aEEG/EEG e hemodinâmico com NIRS) e alterações em achados de imagem incluindo ressonância magnética (RNM) de crânio e ultrassonografia de crânio (transfontanela) realizados durante internação hospitalar.
- Efeitos adversos de hipotermia terapêutica medidos por: arritmia cardíaca, trombocitopenia e distúrbios de coagulação em geral, lesão de pele e hipertensão pulmonar
- Efeito adverso do monitoramento cerebral expresso por lesão de pele decorrente do posicionamento de eletrodos / sensores.
- Associação de achados patológicos de monitoramento cerebral com morbimortalidade e tempo de permanência hospitalar.
- Associação de achados patológicos de monitoramento cerebral com avaliação do neurodesenvolvimento pela aplicação do teste *Bayley* entre 18 e 24 meses de vida.

Coleta de dados (data collection) – os dados de interesse serão coletados por meio do sistema de tecnologia da informação e da central de vigilância e inteligência (CVI) e ainda por avaliação de prontuário do paciente para dados demográficos e resultados de exames de imagem. (Anexo III)

Cálculo amostral (sample size calculation) – A amostra foi definida como não probabilística, por conveniência e de pacientes consecutivos. Considerando a média de quatro pacientes, com critérios para monitoramento neurológico, por mês, em 20 centros, por cinco anos, a previsão é que sejam recrutados aproximadamente 4800 pacientes. O recrutamento será diretamente relacionado à demanda de pacientes internados nos centros de estudo.

Análise dos dados (data analysis) – Os baselines serão analisados por estatística descritiva. As variáveis quantitativas serão apresentadas em média, mediana e desvio padrão e variabilidade dependendo da distribuição e as contínuas especificamente serão analisadas pelos testes t de Student ou Mann-Whitney. Os desfechos primários e secundários serão mostrados em proporção com análise de significância do p, considerando <0,05, com testes de associação de qui-quadrado e Exato de Fisher.

Cronograma do projeto (Timeline of the Project):

**MESES** / **ATIVIDADES**	Out/2020	Novembro/2020 – Dezembro/2020	Janeiro/2021 – Junho/2022	Julho/2022 – Junho/2024	Julho/2024 – Agosto/2024
Revisão da literatura e elaboração do projeto	X				
Submissão à Comissão Científica e Comitê de Ética em Pesquisa	X	X			
Recrutamento de pacientes			X		
Coleta de dados			X		
Avaliação de neurodesenvolvimento de 18-24 meses				X	
Análise dos dados e resultados				X	
Elaboração da discussão e das conclusões do estudo				X	
Elaboração de artigo científico					X
Submissão do artigo em revista científica					X

Orçamento (budget):

O presente projeto de pesquisa será realizado sem gastos adicionais para os centros ou pacientes. Todo o projeto será custeado pela PBSF.

Abreviaturas (Abbreviations)

PBSF: Protecting Brains and Saving Futures

EHI: Encefalopatia hipóxico-isquêmica RN: Recém-nascido
RN: Recém-nascido
UTIN: Unidade de terapia intensiva neonatal
HT: Hipotermia terapêutica
aEEG: Eletroencefalograma de Amplitude Integrada
NIRS: *Near Infrared Spectroscopy*

Referências (references)

1.Kurinczuk JJ, White-Koning M, Badawi N. Epidemiology of neonatal encephalopathy and hypoxic-ischaemic encephalopathy. Early Hum Dev. 2010;86(6):329-338.
2. World health report 2005: Make every mother and child count Geneva: WHO; 2005.
3. Lawn JE, Cousens S, Zupan J. 4 million neonatal deaths: When? Where? Why? Lancet. 2005;365:891–900. doi: 10.1016/S0140-6736(05)71048-5.
4. Lawn JE, Cousens SN, Wilczynska K. Estimating the causes of four million neonatal deaths in the year 2000: statistical annex. In: The world health report 2005 Geneva: WHO; 2005.
5. Shankaran S, Woldt E, Koepke T, et al. Acute neonatal morbidity and long-term central nervous system sequelae of perinatal asphyxia in term infants. Early Hum Dev. 1991;25:135–48.
6. Robertson CMT. Long-term follow-up of term infants with perinatal asphyxia. In: Stevenson DK, Benitz WE, Sunshine P, editors. Fetal and neonatal brain injury. 3rd ed Cambridge University; New York: 2003. pp. 829–58.
7. De Vries LS, Jongmans MJ. Long-term outcome after neonatal hypoxic-ischemic encephalopathy. Arch Dis Child Fetal Neonatal Ed. 2010;95:F220–4.
8. Marlow N, Rose AS, Rands CE, et al. Neuropsychological and educational problems at school age associated with neonatal encephalopathy. Arch Dis Child Fetal Neonatal Ed. 2005;90:F380–7.
9. Blencowe H, Lee ACC, Cousens S, Bahalim A, Narwal R, Zhong N, et al. Preterm birth associated neurodevelopmental impairment estimates at regional and global levels for 2010. Pediatr Res.2013;74(Suppl 1):17–34. doi:10.1038/pr.2013.204
10. Gluckman PD, Wyatt J, Azzopardi DV, et al. Selective head cooling with mild systemic hypothermia after neonatal encephalopathy: multicenter randomized trial. Lancet. 2005;365:663–70.
11. Zhou WH, Cheng GQ, Shao XM, et al. Selective head cooling with mild systemic hypothermia after neonatal hypoxic-ischemic encephalopathy: a multicenter randomized controlled trial in China. J Pediatr. 2010;157:367–72. 372.e1–3.
12. Shankaran S, Laptook AR, Ehrenkranz RA, et al. Whole-body hypothermia for neonates with hypoxic-ischemic encephalopathy. N Engl J Med. 2005;353:1574–84.
13. Azzopardi DV, Strohm B, Edwards AD, et al. Moderate hypothermia to treat perinatal asphyxial encephalopathy. N Engl J Med. 2009;361:1349–58.
14. Simbruner G, Mittal RA, Rohlmann F, et al. Systemic hypothermia after neonatal encephalopathy: outcomes of neo.nEURO.network RCT. Pediatrics. 2010;126(4):e771–8.
15. Jacobs SE, Morley CJ, Inder TE, et al. Whole-body hypothermia for term and near-term newborns with hypoxic-ischemic encephalopathy: a randomized controlled trial. Arch Pediatr Adolesc Med. 2011;165(8):692–700. PMID: 21464374.
16. Tagin MA, Woolcott CG, Vincer MJ, et al. Hypothermia for neonatal hypoxic ischemic encephalopathy: an updated systematic review and meta-analysis. Arch Pediatr Adolesc Med. 2012;166(6):558–66.
17. Edwards AD, Brocklehurst P, Gunn AJ, et al. Neurological outcomes at 18 months of age after moderate hypothermia for perinatal hypoxic ischemic encephalopathy: synthesis and meta-analysis of trial data. BMJ. 2010;9:340–c363.
18. Magalhães, M, et al. Neuroprotective body hypothermia among newborns with hypoxic ischemic encephalopathy: three-year experience in a tertiary university hospital. A retrospective observational study. Sao Paulo Med J. 2015. Jul-Aug;133(4):314-9.
19. Volpe JJ. Neonatal seizures. In: Neurology of the newborn. Philadelphia: WB Saunders; 2008. p. 203e37.

20. Sheth RD, Hobbs GR, Mullett M. Neonatal seizures: incidence, on set and a etiology by gestational age. J Perinatol 1999;19:40e3.
21. Chervenak FA, editors. Fetal and neonatal neurology and neurosurgery. 4th ed. Philadelphia: Elsevier; 2009. p. 698e710.
22. Tekgul H, Gauvreau K, Soul J, et al. The current etiologic profile and neuro-developmental outcome of seizures in term newborn infants. Pediatrics 2006;117:1270e80.
23. Murray DM, Boylan GB, Ali I, Ryan CA, Murphy BP, Connolly S. Defining the gap between electrographic seizure burden, clinical expression and staff recognition of neonatal seizures. Arch Dis Child Fetal Neonatal Ed. 2008;93:F187–91. doi: 10.1136/adc.2005.086314.
24. Abend NS, Wusthoff CJ, Goldberg EM, Dlugos DJ. Electrographic seizures and status epilepticus in critically ill children and neonates with encephalopathy. Lancet Neurol (2013) 12:1170–9.10.1016/S1474-4422(13)70246-1.
25. Hellstrom-Westas L, Rosen I, Svenningsen NW. Predictive value of early continuous amplitude integrated EEG recordings on outcome after severe birth asphyxia in full term infants. Arch Dis Child Fetal Neonatal Ed. 1995;72:F34–8.
26. Thoresen M, Hellström-Westas L, Liu X, de Vries LS. Effect of hypothermia on amplitude-integrated electroencephalogram in infants with asphyxia. Pediatrics. 2010;126:e131–9. doi: 10.1542/peds.2009-2938.
27. Mastrangelo M, et al. Acute neonatal encephalopathy and seizures recurrence: A combined aEEG/EEG study. Seizure. 2013.
28. Shah DK, Zempel J, Barton T, Lukas K, Inder TE. Electrographic seizures in preterm infants during the first week of life are associated with cerebral injury. Pediatr Res. 2010;67:102–6. doi: 10.1203/PDR.0b013e3181bf5914.
29. Payne E.T., Zhao X.Y., Frndova H., McBain K., Sharma R., Hutchison J.S., Hahn C.D. Seizure burden is independently associated with short term outcome in critically ill children. Brain. 2014;137:1429–1438. doi: 10.1093/brain/awu042.
30. van Rooij LG, Toet MC, van Huffelen AC, et al. Effect of treatment of subclinical neonatal seizures detected with aEEG: randomized, controlled trial. Pediatrics 2010;125:e358-66.
31. Vesoulis ZA, Inder TE, Woodward LJ, Buse B, Vavasseur C, Mathur AM. Early electrographic seizures, brain injury, and neurodevelopmental risk in the very preterm infant. Pediatr Res 2014;75:564–9.
32. Srinivasakumar, P, Zempel, J, Trivedi, S, Wallendorf, M, Rao, R, Smith, B and et al. (2015). Treating EEG Seizures in Hypoxic Ischemic Encephalopathy: A Randomized Controlled Trial. Pediatrics 2015136(5): e1302–e1309.
33. Pichler G, Höller N, Baik-Schneditz N,Schwaberger B, Mileder L, Stadler J, Avian A, Pansy J and Urlesberger B. Avoiding Arterial Hypotension in Preterm Neonates (AHIP)—A Single Center Randomised Controlled Study Investigating Simultaneous Near Infrared Spectroscopy Measurements of Cerebral and Peripheral Regional Tissue Oxygenation and Dedicated Interventions. Pediatr. 2018; 6:15.doi: 10.3389/fped.2018.00015.
34. Vesoulis ZA, Lust CE, Liao SM, Trivedi SB, Mathur AM. Early hyperoxia burden detected by cerebral near-infrared spectroscopy is superior to pulse oximetry for prediction of severe retinopathy of prematurity. J Perinatol. 2016; 36(11): 966–971. doi:10.1038/jp.2016.131.
35. Dix LML, van Bel F, Lemmers PMA. Monitoring Cerebral Oxygenation in Neonates: An Update. Pediatr 2017; 5:46. doi: 10.3389/fped.2017.00046.
36. Ministério da Saúde. DATASUS. http://tabnet.datasus.gov.br/cgi/tabcgi.exe?sinasc/cnv/nvuf.def. Acesso 10/01/2018.

37. Hall RW, Hall-Barrow J, Garcia-Rill E. Neonatal Regionalization Through Telemedicine Using a Community Based Research and Education Core Facility. Ethn Dis. 2010; 20(1 0 1): S1–136-40.
38. Burke Jr, Bl. Hall and the section on Telehealth Care. Telemedicine: Pediatric Applications. PEDIATRICS 2015; 136(1):e293-308.
39. McConnochie K, Wood N, Herendeen N, ten Hoopen C, Denk L, Neuderfer J. Integrating telemedicine in urban pediatric primary care: provider perspectives and performance. Telemed J E Health. 2010;16(3):280–288.

ANEXO I (Apendix I) – Protocolo de UTI Neonatal Neurológica

ANEXO II (Apendix II) - Protocolo de Hipotermia Terapêutica

ANEXO III)Appendix III) – Ficha Clínica

ANEXO I

PROTOCOLO UTI NEONATAL NEUROLÓGICA
(Neurocritical care Guidelines)
Protecting Brains & Saving Futures

INTRODUÇÃO (Introduction)

A neonatologia é uma das especialidades médicas que mais evoluíram nas últimas décadas. O incremento do surfactante, óxido nítrico, ventilação protetora promoveram verdadeira revolução na redução da mortalidade neonatal. Entretanto, por diversas vezes esse incremento da sobrevida não foi acompanhado de um neurodesenvolvimento adequado.

Múltiplas são as patologias no período neonatal que estão associadas a alto risco de desenvolvimento de sequelas. Um grupo de destaque é a asfixia perinatal, doença com incidência de 1 a 8 por 1.000 nascidos vivos a termo[1] e que representa a terceira causa mais comum de morte neonatal (23%) após nascimento prematuro (28%) e infecções graves (26%).[2-4] Apesar dos importantes avanços citados nos cuidados perinatais nas últimas décadas, a asfixia continua a ser uma condição grave, e leva a condição denominada encefalopatia hipóxico-isquêmica.

Os recém-nascidos com encefalopatia grave têm um risco muito alto de morte, paralisia cerebral e retardo mental entre os sobreviventes. Os recém-nascidos com encefalopatia moderada apresentam déficits motores significativos, deficiência motora fina, comprometimento da memória, disfunção visual, aumento da hiperatividade e alterações no desempenho escolar.[5-8]

Outra patologia que merece destaque é a prematuridade, com alto risco de sobrevivência acompanhada de déficits neurológicos. Estudos epidemiológicos apontam que no mundo nascem 1,15 milhões de asfixiados e 13 milhões de prematuros ao ano. Destes bebês, 233.000 asfixiados e 350.000 prematuros irão evoluir com sequelas neurológicas moderadas ou graves.[9]

Outras condições clínicas também envolvem riscos importantes de lesão neurológica permanente, destacando-se as situações apresentando na Tabela 1.

Tabela 1. Principais Patologias Associadas a Alto risco de Lesão cerebral Permanente no Período Neonatal

1.	Pacientes com asfixia perinatal / encefalopatia hipóxico-isquêmica (EHI)
2.	Prematuridade Extrema (principalmente se associado a hemorragia peri-intraventricular grave)
3.	Cardiopatia congênita complexa
4.	Malformações cerebrais graves (ex: microcefalia, hidrocefalia)
5.	Infecções congênitas com acometimento de SNC
6.	Sepse / Meningite grave
7.	Erros inatos do metabolismo
8.	Período pós parada cardiorrespiratória prolongada
9.	Crises convulsivas por causas diversas
10.	Instabilidade Hemodinâmica / Ventilatória

Trata-se de uma parcela da população, que quando afetada, demandará de cuidados específicos por toda a vida. Isto representa um impacto social e econômico devastador. Estudos americanos revelam custos durante a vida de crianças com deficiência incapacitante na ordem de U$ 67 bilhões.[10] No Brasil o problema atinge proporções semelhantes e pode ser exemplificado pelo

Benefício da Prestação Continuada (BPC), que tem seu custo em crescimento ascendente com auxílio a pessoas com deficiência incapacitante (2.3 milhões de pessoas). O BPC custou aos cofres públicos em 2015 o montante de R$ 22 bilhões, sendo destes R$ 4.6 bilhões destinados a crianças e adolescentes.[11] O racional para explicar as diferenças nestes custos pode ser explicado pela necessidade de acompanhamento multidisciplinar, aumento expressivo no número de internações, cirurgias e necessidade de medicações especiais.

Considerando que a preservação neurológica é o maior determinante na qualidade de vida de um recém-nascido, a criação de estratégias de avaliação precoce de injúria cerebral é passo fundamental na prevenção de sequelas neurológicas.

Neste cenário, a aplicação de metodologias comprovadamente eficazes para redução de lesão neurológica permanente também tem o potencial de promover a redução de custeio a curto, médio e longo prazo, em casos onde é possível promover desde menor tempo de internação hospitalar do bebê de alto risco, como também redução da necessidade de suporte a crianças com menor severidade de lesão cerebral.

UTI NEONATAL NEUROLÓGICA (Neurological NICU)

O conceito de neurointensivismo já é bem estabelecido em adultos e vem sendo aplicado na faixa etária pediátrica e neonatal mais recentemente. Múltiplos centros norte-americanos e alguns centros brasileiros estão introduzindo o modelo de UTI Neonatal Neurológica, um ambiente altamente especializado composto por equipe multidisciplinar, que provê capacitação de equipe médica e enfermagem, além de implantação de avançadas metodologias para prevenção de sequelas neurológicas na população de risco.[12]

Dentre as metodologias citadas destaca-se a Hipotermia Terapêutica, tratamento específico para pacientes com asfixia perinatal, além de monitoramento cerebral contínuo com eletroencefalografia contínua (EEG) e Near Infrared Spectroscopy (NIRS), aplicável para grupos de recém-nascidos de alto risco.

Em pacientes com encefalopatia hipóxico-isquêmica, diversos estudos foram realizados para avaliação de terapias neuroprotetoras, destacando-se ensaios clínicos randomizados com uso de Hipotermia Terapêutica (HT) demonstrando segurança e eficácia, com redução de morte ou deficiências incapacitantes em lactentes aos 18-24 meses de idade, além de estudos descrevendo experiência bem sucedida na implantação de HT em centros brasileiros.[13-22] Desta forma, há uma recomendação formal em guidelines nacionais e internacionais para o uso de hipotermia terapêutica no tratamento precoce de recém-nascidos com diagnóstico de encefalopatia hipóxico-isquêmica moderada ou grave.[23,24]

Para informações referentes a hipotermia terapêutica, favor consultar o “Protocolo PBSF de Hipotermia Terapêutica pra recém-nascidos com asfixia perinatal”.

Além do benefício terapêutico, é evidenciado um grande benefício econômico ao se prevenir que uma criança evolua com sequelas neurológicas. Estudos clínicos evidenciam custo-efetividade ao promover a aplicação de metodologias comprovadamente eficazes para redução de sequelas neurológicas em recém-nascidos de alto risco.[25]

Devido a comprovada eficácia somada a custo-efetividade, diversos países implantaram em ampla escala o uso das metodologias descritas. O Brasil conta com cerca de 3 milhões de nascidos vivos por ano e um alto número estimado de bebês com alto risco para injúria cerebral, incluindo recém-nascidos com EHI, prematuros, nascidos com cardiopatia congênita, malformações cerebrais, sepse

grave entre outras condições de risco.[26] Apesar do benefício descrito, ainda estima-se que menos de 5% dos centros neonatais brasileiros utilizam hipotermia terapêutica ou monitoramento cerebral contínuo de forma rotineira, protocolizada e bem estruturada para o atendimento de recém-nascidos de alto risco.[27]

Eletroencefalograma de Amplitude Integrada (Amplitute Integrated EEG)

O eletroencefalograma de amplitude integrada (aEEG) é uma técnica de registro eletroencefalográfico que utiliza 1 a 3 canais onde ocorre a compressão das amplitudes mínima e máxima permitindo avaliação da tendência do EEG ao longo das horas.[28]

Essa metodologia possibilita monitorização cerebral contínua a beira leito em recém-nascidos de alto risco, fornecendo informações sobre a função cerebral em tempo real além de ter papel fundamental na detecção precoce de crises epilépticas.[28,29] O aEEG pode ser associado a leitura de EEG bruto e vídeo imagem (vídeo aEEG/EEG), aumentando desta forma a sensibilidade e especificidade para avaliação de crises epilépticas.[30-33] Dentro da avaliação de função cerebral, há evidências mostrando associação de padrão patológico persistente com pior neurodesenvolvimento em pacientes com asfixia perinatal, prematuridade extrema e cardiopatas congênitos.[34-36]

Estudos clínicos demonstram grande aplicabilidade clínica, permitindo avaliação prognóstica e neurológica atual em recém-nascidos de alto risco.[37,38] Alterações visualizadas de forma dinâmica na atividade de base e atividade epiléptica estão relacionadas a função e injúria cerebral, podem ser interpretadas em tempo real e permitem ações muito mais rápidas e assertivas em relação ao quadro clínico vigente.

Montagem (Assembly)

No protocolo PBSF é utilizado a montagem do aEEG em 3 canais, associado ao eletroencefalograma bruto e monitorização com vídeo (Vídeo aEEG/EEG). Os três canais utilizados de rotina são C3-P3, C4-P4 e P3-P4, posicionados de acordo com o Sistema Internacional 10-20%.[39]

Por convenção, os pontos pares estarão posicionados à direita e os ímpares à esquerda. Deve-se atentar para evitar posicionamento dos eletrodos sobre lesões de pele, região de suturas cranianas e áreas com edema ou hematoma importante.

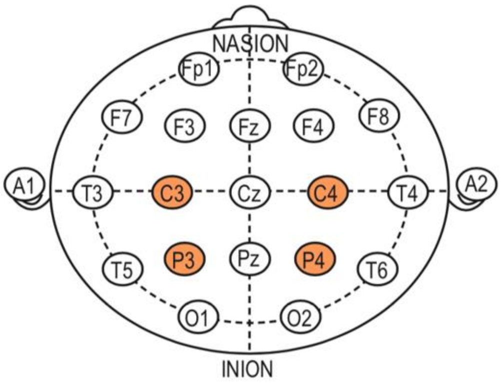

Figura 1. Sistema Internacional 10-20% para posicionamento de eletrodos de eletroencefalograma.

Indicações de Monitorização (Indications for monitoring)

As principais indicações de monitoramento estão descritas na Tabela 2. As indicações que não

se enquadrem nas citadas abaixo poderão ser discutidas individualmente entre equipe assistencial de neonatologia, neurologia e equipe de assistência remota da PBSF.

A duração do monitoramento dependerá da condição clínica do paciente, porém recomenda-se aguardar o tempo mínimo de 24 horas após controle total das crises epilépticas para suspensão da monitorização.

Em pacientes asfixiados recomenda-se iniciar a monitorização o mais precoce possível, idealmente nas primeiras 6 horas de vida. Em pacientes com indicação de hipotermia terapêutica, manter durante todo o período de resfriamento, com suspensão após 24 horas do término do reaquecimento. Nos pacientes que não tenham critérios para hipotermia terapêutica, sugere-se monitorizar por 24 horas na ausência de crises epilépticas.

Tabela 2. Principais Indicações de Monitoramento com Vídeo aEEG/EEG.

	**Indicações de Monitoramento**
**1**	EHI moderada /grave
**2**	EHI leve
**3**	Acidente Vascular Cerebral
**4**	Crise Convulsiva Prévia ou Suspeita
**5**	Prematuridade extrema
**6**	Instabilidade hemodinâmica/ ventilatória
**7**	Hemorragia Peri-intraventricular grave
**8**	Erros Inatos do Metabolismo
**9**	Cardiopatia Congênita Complexa
**10**	Malformação grave de SNC
**11**	Hérnia Diafragmática
**12**	Infecção de SNC ou Sepse grave
**13**	Hiperbilirrubinemia grave
**14**	ECMO

Central de Monitoramento Remoto (Remote monitoring center)

Os dados gerados pelo exame serão enviados a uma central de monitorização inteligente denominada Central de Vigilância e Inteligência (CVI), com servidores capazes de armazenar os dados de forma segura, que redirecionará os dados a equipe médica para avaliação contínua.

Esta central também terá um papel de banco de dados que promoverá interação estatística, que virá a ser uma importante estratégia para desenvolvimento de estruturação de banco de dados e pesquisas científicas.

Monitoramento Remoto por Equipe Médica (Remote monitoring by the medical team)

A partir do momento em que é instalado o equipamento e iniciado o registro do exame, um médico responsável inicia a monitorização por acesso remoto de forma contínua. O médico pode

entrar em contato diretamente com a equipe médica para discussão do caso, orientar diagnósticos, sugerir intervenções e tratamentos a fim de promover homogeneidade do cuidado neonatal. Deve realizar o mínimo de 4 evoluções diárias.

Cabe à equipe médica remota:

- Poder entrar em contato ou ser contatado a qualquer momento pela equipe assistencial à beira leito. Desta forma, alterações como atividade epiléptica subclínica e depressão súbita da atividade elétrica cerebral serão comunicadas abrindo a possibilidade para intervenção precoce.
- Comunicar-se com a equipe da UTI para discussão do caso e explicar à equipe médica os achados do exame sempre que necessário.
- Ao final do período de registro emitirá um laudo com o resultado do exame.

Interpretação do aEEG (aEEG interpretation)

1) Atividade elétrica cerebral de base

A atividade elétrica cerebral de base pode ser utilizada na avaliação de função cerebral em tempo real, sendo descritos 5 padrões existentes.(37

Alterações no padrão de atividade elétrica de base ao longo do tempo, em conjunto com os achados clínicos, podem auxiliar no diagnóstico e manejo em diferentes situações, permitindo avaliação objetiva de injúria cerebral.

a) Padrão de voltagem Contínuo: amplitude mínima de pelo menos 5μV e amplitude máxima de pelo menos 10μV, geralmente entre 10 - 50μV.

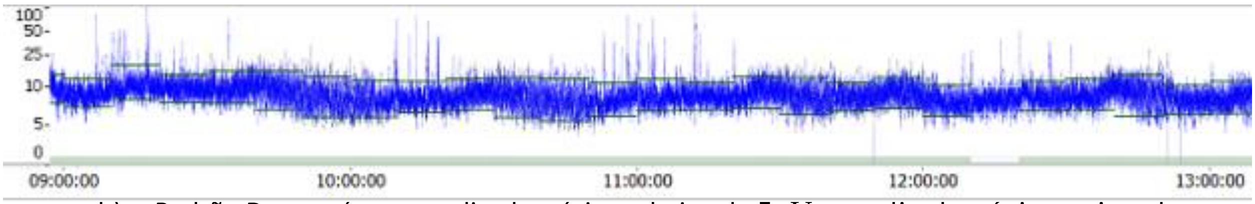

b) Padrão Descontínuo: amplitude mínima abaixo de 5μV e amplitude máxima acima de 10μV. O padrão descontínuo ainda pode ser subclassificado em:
   I. Descontínuo de alta voltagem: amplitude mínima entre 3 a 5μV e amplitude máxima acima de 10 μV.
   II. Padrão de voltagem Descontínuo: amplitude mínima abaixo ou igual a 5μV e amplitude máxima acima de 10 μV.

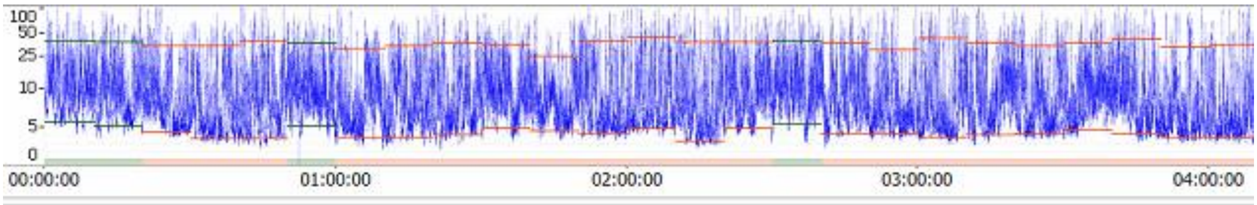

Figura 3: Padrão de voltagem Descontínuo. Arquivo pessoal do autor, [illegible]

c) Padrão em Surto Supressão: amplitude mínima entre 0 a 1 μV, sem variabilidade, e com surtos de amplitude acima de 25μV.

O padrão em surto supressão ainda pode ser dividido entre:
   I. SS + indica densidade de surtos acima de 100 surtos por hora.

II. SS - indica densidade de surtos abaixo de 100 surtos por hora.

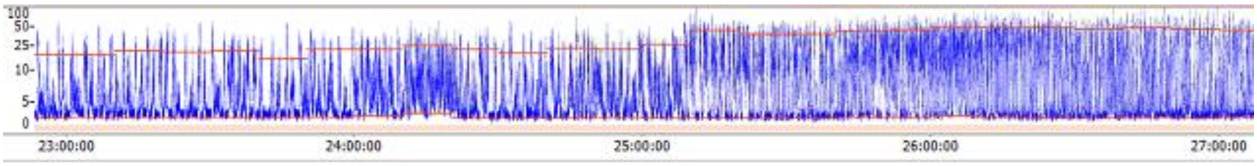

d) Padrão Contínuo de baixa voltagem: amplitude mínima abaixo de 5 µV e amplitude máxima abaixo de 10µV.

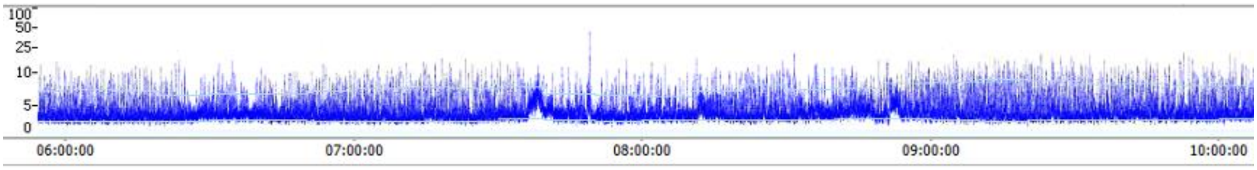

e) Padrão Isoelétrico: amplitude máxima e mínima continuamente abaixo de 5 µV.

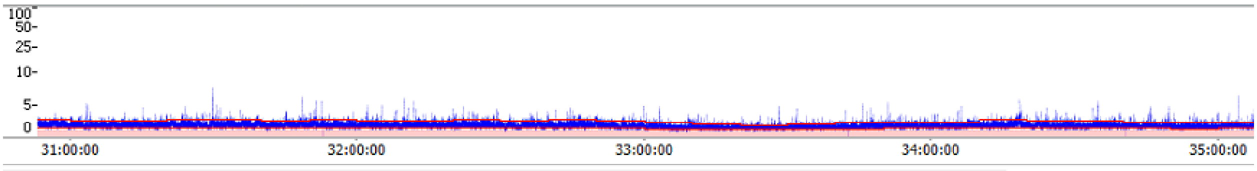

Nos bebês a termo, o padrão de voltagem Contínuo é considerado o padrão de normalidade. Em prematuros extremos o padrão primeiramente encontrado será o Descontínuo, evoluindo progressivamente para padrão Contínuo com o aumento da idade gestacional ao longo das semanas. Os padrões Contínuo de baixa voltagem, Surto Supressão e Isoelétrico são padrões considerados patológicos, independentemente da idade gestacional do paciente.[37]

Em casos de traçado assimétrico, deve-se avaliar o posicionamento correto dos eletrodos e a presença de edema ou hematoma unilateral. Após descartar essas causas, se a assimetria do traçado persistir, recomenda-se realizar exame de imagem para pesquisa de lesões unilaterais de sistema nervoso central.

2) Ciclo Sono vigília

O ciclo sono e vigília visualizado através do aEEG é caracterizado por variações cíclicas das bandas de amplitude. Períodos onde as bandas de amplitude apresentam maior largura representam a atividade mais descontínua durante o sono profundo e tem duração de pelo menos 20 minutos. Já os períodos onde as bandas de amplitude se aproximam, tornando o traçado mais estreito, correspondem ao momento de vigília ou sono superficial.[28]

Em prematuros extremos, a presença de ciclo sono vigília imaturo pode ser identificada a partir de 25 semanas de idade gestacional. A partir de 31 semanas, o ciclo sono vigília já pode se tornar claramente identificável. Nos bebês a termo espera-se encontrar ciclo sono vigília completamente desenvolvido.[28,37]

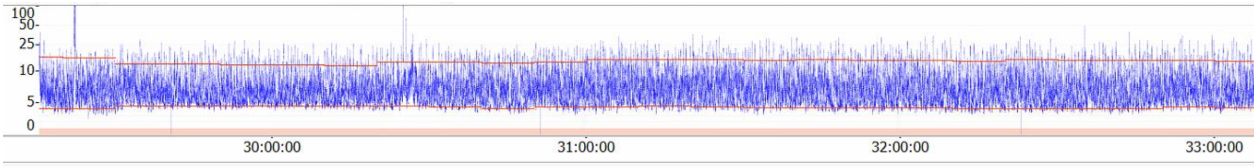

Figura 7. Representação de ciclo sono vigília ausente no aEEG. Arquivo pessoal do autor, 2020.

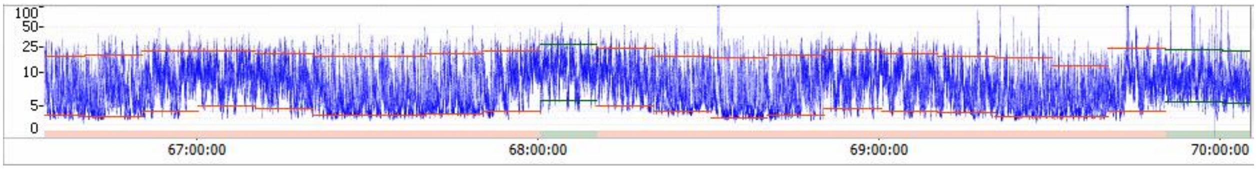

Figura 8. Representação de ciclo sono vigília imaturo no aEEG. Arquivo pessoal do autor, 2020.

Figura 9. Representação de ciclo sono vigília desenvolvido no aEEG. Arquivo pessoal do autor,2020.

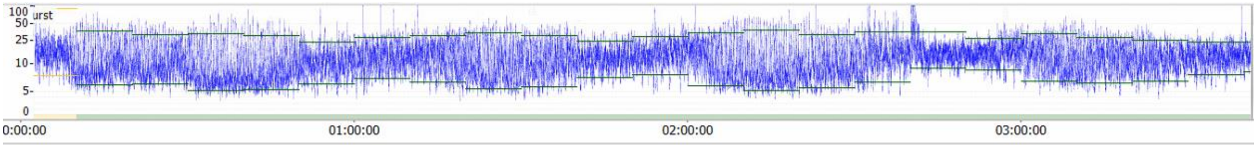

3) Crises Epilépticas

A crise epiléptica pode ser visualizada ao aEEG através da elevação das amplitudes do traçado. Manipulação do paciente e diversos artefatos podem mimetizar o mesmo achado durante a leitura do aEEG, por isso é de fundamental importância associar a visualização do EEG bruto de forma simultânea para comprovação da crise epiléptica.[30] Além do aEEG e EEG bruto, associa-se a vídeo-gravação contínua, que também auxilia na detecção de artefatos e manipulações.

No EEG bruto, a crise epiléptica é caracterizada por evento eletrográfico anormal que se destaca da atividade de base e é bem definido, com início e término claros, consistindo de ondas agudas/espículas ou ondas rítmicas repetitivas, sustentadas e evolutivas em amplitude, frequência e localização, com duração mínima de 10 segundos.[40]

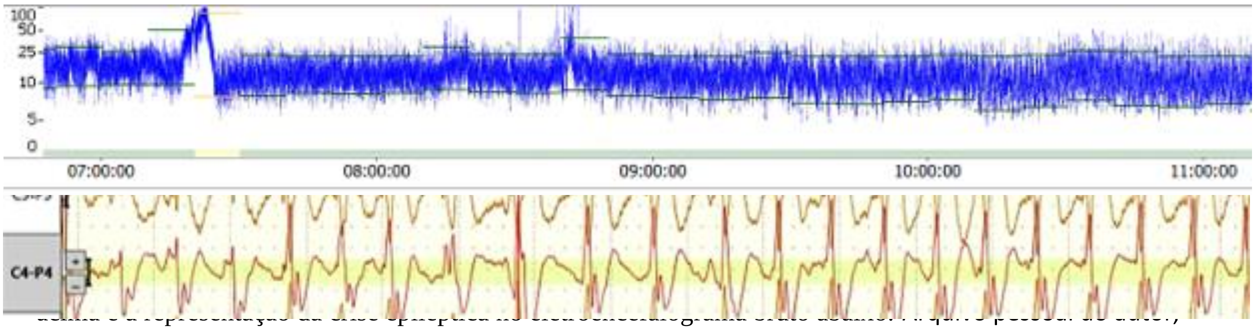

[illegible]
2020.

Principais Utilidades Clínicas (Main Clinical Indications)

<u>Convulsão Neonatal</u> (Neonatal Seizures)

A incidência de crises convulsivas é maior no período neonatal do que em qualquer outro período da vida e estão em sua maioria, atribuídas a injúria cerebral aguda.[41]

O eletroencefalograma convencional realizado de forma contínua é considerado o padrão ouro no diagnóstico de crises epilépticas neonatais, porém não se trata de metodologia de fácil acesso. O aEEG surge então como uma opção interessante, porém com sensibilidade e especificidade inferiores. Dessa forma, o uso do aEEG contínuo associado a leitura do EEG bruto aumentou a acurácia principalmente quando interpretado por profissional experiente sendo capaz de detectar 100% dos casos de mal epiléptico subclínicos e a grande maioria das crises epiléticas repetitivas.[30-33]

Estudos revelam que mais de 80% das crises epilépticas e até mesmo estados de mal epiléptico dentro da UTI neonatal são completamente subclínicos. Por outro lado, suspeitas clínicas de convulsão frequentemente não correspondem a eventos eletroencefalográficos, podendo levar a administração desnecessária de anticonvulsivantes e sua repercussão.[42,43] Portanto o diagnóstico clínico de convulsão neonatal pode ser extremamente falho.

Quanto à necessidade de tratamento, já foi demonstrado em estudos prévios que o tratamento das crises epilépticas subclínicas esteve associado a redução do tempo total de atividade epiléptica e melhor neurodesenvolvimento.[44,45] Além disso, unidades que dispõem de monitorização eletrográfica, apresentam menor uso de anticonvulsivantes, considerando menor dose total utilizada de fenobarbital e o menor número de pacientes que recebem alta com anticonvulsivantes, refletindo a redução de medicações anticonvulsivantes desnecessárias nas manifestações clínicas que não se confirmam na avaliação eletrográfica, além do manejo mais assertivo com o uso da monitorização contínua a beira leito.[46,47]

<u>Asfixia Perinatal</u> (Perinatal Asfixia)

O uso do Vídeo aEEG/EEG em pacientes com encefalopatia hipóxico-isquêmica moderada ou grave tem diversas utilidades, incluindo avaliação de função cerebral em tempo real, acompanhamento da recuperação da atividade cerebral de base após o insulto agudo com valor prognóstico e detecção de crises epilépticas.[48,49]

Nos recém-nascidos com EHI limítrofe entre leve e moderada, a monitorização pode ser particularmente útil auxiliando na indicação de HT em conjunto com os achados clínicos e laboratoriais, considerando que o padrão de atividade de base patológico e/ou a presença de crises epilépticas são achados indicativos de injúria cerebral importante.[50]

Existem estudos comprovando relação prognóstica com o uso do aEEG na asfixia perinatal. Com a monitorização contínua, pode-se avaliar o Tempo para Traçado Normal (TTN) que será o tempo necessário, em horas de vida, para normalização da atividade cerebral de base. Nos recém-nascidos submetidos a HT, atividade de base patológica até 48 a 72 horas de vida apresenta alto valor preditivo de morte ou pior desfecho neurológico a longo prazo.[51]

Quanto à avaliação de crises epilépticas, a asfixia perinatal representa a principal causa de crises no período neonatal, representando de 40 a 60% da etiologia de crises em bebês a termo. As crises epilépticas nesse grupo de pacientes ocorrem mais frequentemente no primeiro dia de vida e no período de reaquecimento[52], por isso indica-se a monitorização eletrográfica o mais precoce possível após o nascimento e suspensão apenas após 24 horas do término do reaquecimento.

A presença de crises epilépticas é fator isolado de risco para atrasos no neurodesenvolvimento e o reconhecimento e tratamento imediato reduz a sua duração e tem relação com melhor neurodesenvolvimento.[44,45,53]

Prematuridade extrema (Extremely prematurity)

Em prematuros extremos, o aEEG mantém sua utilidade na avaliação da função cerebral em tempo real, permitindo diagnosticar injúria cerebral precocemente principalmente em casos de instabilidade ventilatória ou hemodinâmica, frequentemente observadas na primeira semana de vida. Além disso, a incidência de crises epilépticas em prematuros extremos pode ser elevada, sendo descrita incidência variando de 5 a 48% em estudos prévios.[54-58]

O aEEG apresenta boa relação prognóstica quando avaliado nos primeiros dias ou semanas de vida. Estudos demonstram que alterações eletrográficas nessa idade, como atividade de base patológica, ausência de ciclo sono vigília e presença de crises epilépticas, apresentam relação com maior risco de morte, hemorragia peri-intraventricular grave, leucomalácia periventricular e pior desfecho neurológico a longo prazo. A sensibilidade e especificidade do aEEG para os desfechos citados foram superiores quando comparados à ultrassonografia de crânio.[54-58]

Cardiopatia Congênita (Congenital Heart Disease)

Em pacientes com cardiopatia congênita complexa, o monitoramento com aEEG tem grande utilidade no reconhecimento precoce de injúria cerebral. Alterações eletrográficas são vistas frequentemente nessa população, seja no período pré-operatório, intraoperatório ou pós-operatório, geralmente relacionados a alterações de perfusão ou oxigenação cerebral.[59]

Estudos revelam alta incidência de crises epilépticas, na sua maioria subclínicas. Além disso, também foi encontrada associação entre recuperação precoce da atividade de base e presença de ciclo sono vigília após a cirurgia com bom desfecho neurológico. Por outro lado, a presença de padrão eletrográfico patológico por mais de 48 horas após a intervenção cirúrgica esteve associada a pior evolução neurológica em relação a escore motor e cognitivo.[59] Além disso, padrão patológico por mais de sete dias de pós operatório foi associado com alto risco de morte.[60]

Outras Situações Clínicas (Others)

O aEEG também é útil na monitorização de outros grupos de pacientes para avaliação de função cerebral e de crises epilépticas, incluindo distúrbios metabólicos, erros inatos do metabolismo, acidente vascular cerebral, malformações de sistema nervoso central, sepse, meningite, infecções congênitas com acometimento neurológico, instabilidade hemodinâmica ou ventilatória e ECMO.

Olichar et al. avaliaram 30 crianças com erros inatos do metabolismo e encontraram atividade de base patológica em 70% e presença de crises epilépticas em 60%. Lactentes com distúrbios do metabolismo energético, hiperamonemia e aminoacidopatias apresentaram depressão importante da atividade cerebral de base com crises epilépticas, em contraste com pacientes com distúrbios peroxissômicos que não apresentaram alterações importantes na monitorização cerebral.[61]

Toso et al. avaliaram 21 recém-nascidos com diferentes causas de encefalopatia, distúrbios neurológicos ou síndrome do desconforto respiratório grave e encontraram 38% de incidência de crises epilépticas.[38]

Helderman et al. estudaram 108 pré-termos extremos, realizando aEEG mensalmente, de 28 semanas a 36 semanas de idade gestacional corrigida. Monitorizações adicionais foram realizadas no primeiro episódio de sepse destes pacientes. Os autores encontraram presença de atividade de base patológica em 22% dos prematuros sem diagnóstico de sepse e em 57% dos prematuros com sepse atual, concluindo haver associação entre sepse e alterações eletroencefalográficas agudas.[62]

Alguns medicamentos, como sedativos e medicações anticonvulsivantes, podem deprimir a atividade elétrica cerebral de base vista no aEEG, variando conforme a medicação e dose utilizada. Outra situação que frequentemente cursa com alteração da atividade cerebral de base é a administração de surfactante, podendo levar a depressão transitória por cerca de 10 minutos após a dose.[63]

Near Infrared Spectroscopy

A Espectroscopia de infravermelho próximo é uma metodologia não invasiva para avaliação da saturação regional de oxigênio (rSO2) de forma contínua a beira leito.

Essa ferramenta funciona através da emissão de luz na faixa do infravermelho próximo, que penetra nos tecidos moles e nos ossos, em particular tecidos finos e no crânio, atingindo cromóforos em circulação, que são substâncias capazes de absorver e transmitir de modo modificado esse infravermelho próximo dependendo do status de oxigenação destes. Os cromóforos que podem ser mensurados incluem a hemoglobina (Hb). Alterações na concentração tecidual de oxiemoglobina (HbO2) e desoxiemoglobina (HHb) são medidas em tempo real em uma razão de 25% em sangue arterial e 75% em sangue venoso.[64-67]

Com o uso do NIRS podemos extrair duas informações:

- Saturação Regional (rSO2), expressa em porcentagem que *reflete o equilíbrio regional entre o suprimento de oxigênio e o consumo pelo tecido/órgão adjacente*, e é dada pela fórmula:[68]

$$rSO2 = \frac{HbO2}{HbO2 + HHb}$$

Onde:
- rSO2 = Saturação Regional de Oxigênio
- HbO2 = Oxiemoglobina
- HHb = Desoxiemoglobina

Portanto, a rSO2 reflete diretamente o balanço entre oferta e demanda de oxigênio tecidual onde:

a) A rSO2 será mais alta se houver maior oferta ou menor consumo de O2.
b) A rSO2 será mais baixa se houver menor oferta ou maior consumo de O2.

- Fração Tecidual de Extração de Oxigênio (FTOE), expressa em decimais que *reflete o consumo de oxigênio em relação à entrega de oxigênio ao tecido,* e é dada pela fórmula.[68]

$$FTOE = \frac{SaO2 - rSO2}{SaO2}$$

Onde:
- FTOE = Fração Tecidual de Extração de Oxigênio
- rSO2 = Saturação Regional de Oxigênio
- SaO2 = Saturação Arterial de Oxigênio

Portanto, o monitoramento com NIRS permite a mensuração em tempo real, à beira do leito, de oxigenação regional do tecido cerebral e somático e pode fornecer informações úteis sobre estado hemodinâmico e risco de lesão cerebral.

A saturação regional tecidual (rSO2) reflete o equilíbrio entre a entrega e o consumo de oxigênio no tecido subjacente, sendo expressa em porcentagem. A informação é disponibilizada em tempo real, com leitura a cada 2 a 5 segundos e a tendência ao longo do tempo pode ser demonstrada.[66]

Posicionamento e Sítios de Monitoramento (Placement and monitoring sites)

Para garantir um bom contato do sensor, é necessário limpar, desengordurar e secar adequadamente a pele antes do posicionamento.

- Sensor cerebral:

O sensor cerebral pode ser posicionado no centro, lado direito ou esquerdo da fronte.[66]

Posicionar o sensor sobre os cabelos, hematomas ou edemas pode causar leituras imprecisas, erráticas ou incapacidade de leitura.

- Sensor somático:

- Renal: em flanco posterior entre T10 e L2 (abaixo do último arco costal e acima da crista ilíaca), à direita ou esquerda da linha média [(66)].
- Mesentérica: Abaixo da cicatriz umbilical, na linha média [(66)].

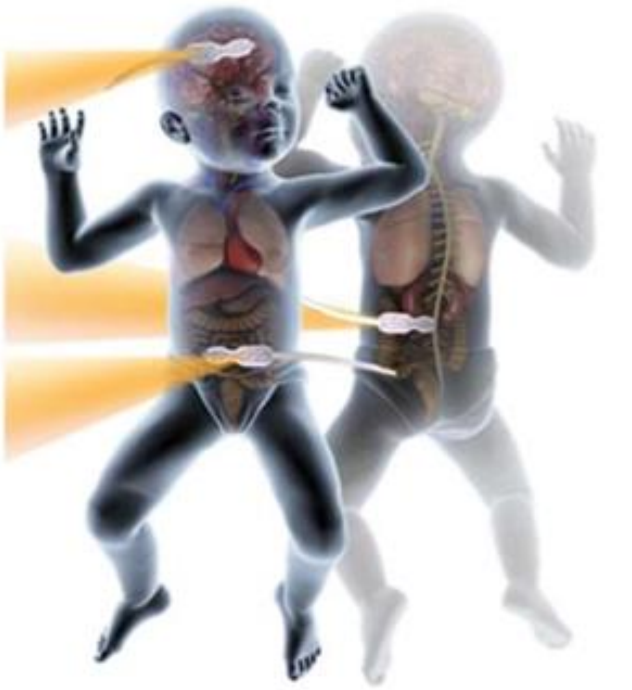

Figura 11. Ilustração do posicionamento dos sensores do NIRS. Fonte: Somanetics Corporation. INVOS® Cerebral/Somatic Oximeter Quick Reference Guide for Pediatric Use. Troy, Michigan, USA.

Em neonatos, pode-se realizar monitoramento cerebral e somático simultâneo, uma vez que é possível obter informações comparativas das mudanças da oxigenação e perfusão entre circulação cerebral e somática.[69]

a) Saturação regional cerebral
    - A saturação regional cerebral (rScO2) é monitorada para avaliar precocemente insultos cerebrais em condições que afetam a perfusão e oxigenação cerebral.

- O sistema nervoso central apresenta alta demanda metabólica culminando em alta taxa de extração de oxigênio, portanto os valores de rScO2 tendem a ser menores.
- Além disso, o cérebro também apresenta mecanismos compensatórios, como autorregulação cerebral, portanto, a variabilidade da rScO2 ao longo do tempo tende a ser menor.
- Destaca-se que a perda de autorregulação cerebral e a queda da rScO2 representa um indicador <u>tardio</u> de instabilidade hemodinâmica e choque.

b) Saturação regional somática
- A rSO2 somática, renal (rSrO2) ou mesentérica (rSmO2) é monitorada para avaliar de forma precoce alterações de oxigenação tecidual nestes sítios.
- A demanda metabólica somática é menor em comparação com a demanda cerebral, portanto os valores de rSrO2/rSmO2 tendem a ser maiores.
- O fluxo sanguíneo somático é variável e regulado principalmente pelo sistema nervoso simpático. A queda da saturação somática, em especial a saturação regional renal, é considerada um indicador <u>precoce</u> de instabilidade hemodinâmica e choque.

Vale ressaltar as várias limitações significativas relacionadas ao monitoramento do NIRS mesentérico. Ao contrário do tecido cerebral, o distrito mesentérico inclui várias estruturas diferentes (por exemplo, intestino grosso e delgado, ureteres e bexiga); e é, portanto, desafiador definir exatamente a contribuição de cada tecido. Além das variáveis acima, os movimentos intestinais devidos ao peristaltismo fisiológico também podem aumentar o ruído do sinal NIRS abdominal e tornar a monitorização de rSmO2 bastante propensa a artefatos. Ao contrário da rScO2, a monitorização mesentérica mostrou variabilidade individual significativa na população neonatal, resultando em padrões flutuantes que podem dificultar não apenas o estabelecimento de valores de referência, mas também o papel dessa técnica de monitoramento para guiar decisão clínica.[70]

Aplicabilidade Clínica (clinical indications)

A aplicabilidade do NIRS vem sendo demonstrada em diversos grupos, sendo os principais:

<u>Asfixia Perinatal</u>

Vários estudos de coorte em bebês com EHI encontraram associação entre achados de monitoramento por eletroencefalograma de amplitude integrada (aEEG) e também da espectroscopia de infravermelho próximo (NIRS), como importantes preditores precoces de neurodesenvolvimento a longo prazo após asfixia perinatal.

Recém-nascidos asfixiados que evoluíram com grave alteração no neurodesenvolvimento no futuro, tiveram mais frequentemente entre 12 e 24 horas de vida, um aumento anormal da rScO2. A falha energética secundária nesta população fica evidenciada com uma redução no consumo de oxigênio pelas células neuronais gravemente lesadas, e uma rScO2 supranormal às 24 horas de vida pode ser explicada pelo baixo metabolismo após lesão cerebral grave, indicativa de baixa utilização de oxigênio, hiperperfusão cerebral e autorregulação prejudicada. Esse achado tem sido associado à morte e a resultados adversos no desenvolvimento neurológico. [71]

Lemmers et al, estudaram o valor prognóstico de aEEG e NIRS em 39 RN a termo com EHI. A partir de 24 horas de vida, a rScO2 encontrada foi significativamente maior no grupo com resultados desfavoráveis. Ambos o aEEG e a rScO2 foram preditores de resultados associados a bom neurodesenvolvimento, mas menos confiáveis para prever resultados adversos. Os autores mostraram que, em comparação com cada modalidade isolada, a combinação de dados NIRS e aEEG melhorou entre 12 a 18 horas de vida o valor preditivo positivo (NIRS 67% e aEEG 62% versus 91% combinado) e o valor preditivo negativos (NIRS 73% e aEEG 100% versus 100% combinado).[72]

Com as evidências atuais, o NIRS se mostra útil no manejo do paciente com asfixia perinatal e com valor prognóstico principalmente quando associado ao aEEG.

Prematuros extremos

As primeiras horas de vida são de extrema importância, por se tratar de período de transição do sistema circulatório e respiratório associado à imaturidade e a falta de autorregulação do fluxo sanguíneo cerebral, portanto estão sob alto risco de hipóxia e/ou hiperóxia, hipotensão sistêmica associada a hipofluxo cerebral e aumento da chance de hemorragia peri-intraventricular (HPIV).

O consórcio SefeBoosC concluiu recentemente um estudo controlado randomizado de fase II, estudando bebês extremamente prematuros nas primeiras 72 horas de vida e demonstrou a viabilidade e eficácia da implementação do monitoramento contínuo NIRS com uma diretriz de tratamento. Os bebês do grupo monitorado pelo NIRS apresentaram menos episódios de hipóxia ou hiperóxia cerebral em comparação com o grupo controle, além de tendência a menor mortalidade e redução de HPIV grave.[73]

Em uma coorte de 68 prematuros, Vesoulis et al. encontraram associação da FTOE, mas não da SpO2, com retinopatia grave da prematuridade (ROP), sugerindo que o NIRS pode ser uma ferramenta melhor para reduzir a ROP do que a SpO2.[74]

Quanto a persistência do canal arterial (PCA) com repercussão hemodinâmica, é uma patologia comum entre os RN prematuros extremos, chegando a acometer aproximadamente 70% dos casos. O fluxo da esquerda para a direita afeta a perfusão e a oxigenação cerebral e renal, apresentando menor saturação regional e maior risco para hemorragia intraventricular e lesão na substância branca. Chock et al. realizou um estudo comparando a saturação regional cerebral e renal de RN com PCA com repercussão hemodinâmica com a de RN sem PCA ou com PCA sem repercussão. Os autores concluíram que valores baixos da saturação renal (rSrO2 <66%) estavam associados com a presença de PCA com repercussão hemodinâmica. Dessa forma, a monitorização com NIRS auxilia também na suspeita de PCA.[75]

Cardiopatia Congênita

O dano neurológico em pacientes com cardiopatia congênita complexa pode ocorrer desde a vida intrauterina e mais de 50% desses pacientes apresentam deficiências no neurodesenvolvimento a longo prazo devido às alterações de fluxo e oxigenação causadas pelas malformações cardíacas.

O racional para o uso de NIRS em pacientes com cardiopatia congênita envolve os seguintes aspectos:

- Independentemente da oxigenação sistêmica (SpO2), a oxigenação cerebral ou somática pode ser inadequada;
- Os valores de NIRS de correlacionam de forma precoce com outros indicadores de má perfusão sistêmica como lactato elevado, perfusão periférica alterada e baixo débito urinário;
- O uso de NIRS pode auxiliar para avaliar a eficácia ou necessidade de intervenções adicionais como alterações no suporte ventilatório, diuréticos, alteração na dose de prostaglandina, necessidade de transfusão sanguínea e intervenção cirúrgica precoce.

Abaixo descrevemos alguns estudos e exemplos de cardiopatia congênita onde o uso de NIRS foi avaliado:

NIRS e período perioperatório de cardiopatias complexas:

Dodge-Kathami et al. conduziram um estudo prospectivo com o objetivo de avaliar o valor preditivo da monitorização com NIRS para eventos adversos clínicos no período perioperatório de cardiopatias complexas. 41 pacientes foram monitorizados com sensor NIRS cerebral e renal, os valores foram mensurados em 20 momentos. As conclusões do estudo foram de que a saturação cerebral e renal no período perioperatório, respectivamente, menores que 45% e 40% estavam correlacionadas à necessidade de circulação extracorpórea (ECMO) e óbito, e a saturação renal menor que 30% estava correlacionada com aumento no tempo de internação na UTI.[76]

NIRS e insuficiência renal após intervenção cirúrgica:
Pacientes submetidos a cirurgias cardíacas são considerados de alto risco para insuficiência renal, e esta por si só, aumenta o risco de mortalidade. Uma coorte prospectiva realizada por Colasacco et al. avaliou 48 pacientes submetidos a cirurgia cardíaca no período intraoperatório e pós-operatório; os valores do NIRS, diurese, balanço hídrico e creatinina sérica foram avaliados em 3 períodos. Os autores concluíram que a monitorização intra e pós-operatória com NIRS pode prover um marcador precoce e não invasivo de insuficiência renal após cirurgias cardíacas em lactentes, uma vez que a saturação renal pode prever a insuficiência renal com sensibilidade de 100% e especificidade de 75%. Esses resultados podem ser clinicamente significantes se forem realizadas intervenções terapêuticas a fim de melhorar a saturação e função renal, com o objetivo de prevenir a falência renal.[77]

NIRS e desfecho clínico precoce:
Estudos têm mostrado que com a monitorização com NIRS é possível prever desfechos iniciais como óbito e necessidade de ECMO, bem como em pacientes submetidos a ECMO os valores da monitorização com NIRS podem ser considerados fortes fatores prognósticos de sobrevivência e de presença de lesão cerebral.[78-79]

Choque (shock)

O diagnóstico precoce de choque é crucial para o início de tratamento rápido e eficaz. Vários índices relacionados à oxigenação são utilizados como preditores de choque, como consumo de oxigênio (VO2), entrega de oxigênio (DO2), lactato, saturação de oxigênio (SpO2), saturação venosa central (ScvO2), saturação arterial de oxigênio (SaO2) e pressão parcial de oxigênio (PO2). Dentre todos estes citados, apenas a SpO2 pode ser detectada através de método não invasivo.

Neste contexto, o uso do NIRS permite uma monitorização não invasiva e contínua da oxigenação tecidual regional. Existem diversos estudos usando o NIRS como preditor de choque em adultos, crianças e recém-nascidos. Um destes estudos demonstrou uma relação significante em 25 pacientes entre rScO2 e a ScvO2, assim como outros marcadores de oxigenação, dando suporte para o NIRS como marcador não invasivo de choque.[80]

Em recém-nascidos, a incidência de hipotensão é inversamente proporcional à IG e peso de nascimento. Para hipotensão arterial, diversas definições são usadas, sendo as mais comuns a pressão arterial média (PAM) menor que 30mmHg ou menor que a idade gestacional, expressa em mmHg.

Apesar desta definição ser amplamente utilizada e muito aceita, há diversos estudos que não acharam associação de hipotensão arterial e alterações de neurodesenvolvimento futuro.

Baseado na premissa, acima citada aliado ao racional de que o monitoramento NIRS poder ser um preditor precoce de choque, Alderliesten et al.[81] avaliaram uma coorte de 66 prematuros com menos de 32 semanas de IG ao nascimento, com diagnóstico de choque em uso de dopamina e/ou outro medicamento inotrópico para tratamento de choque, excluindo pacientes com diagnóstico de persistência de canal arterial. Foram monitorizadas a PAM e a rScO2 de todos os indivíduos do estudo durante o tratamento de choque e comparados o neurodesenvolvimento com 18 a 24 meses de idade corrigida. Os autores concluíram que ter uma PAM menor que a IG ou menor do que 30mmHg de forma isolada não teve associação com desfecho neurológico; porém aqueles indivíduos que tiveram rScO2 menor que 55% por período prolongado, independentemente da PAM, tiveram associação com pior desfecho neurológico.[81]

Neste contexto, estudos estimam que a rSO2 seja o biomarcador mais precoce de alteração de perfusão e oxigenação tecidual.

Anemia (Anemia)

A anemia é a doença hematológica mais comum no período neonatal, em especial na população prematura. Dentre as causas de anemia relacionadas a prematuridade estão a produção inadequada de eritropoietina, menor tempo de vida de hemácias e espoliação devido à frequente coleta de exames na UTI.

O momento e a necessidade de uma transfusão de concentrado de hemácias residem como

pontos de grande questionamento. Temos conhecimento sobre a necessidade de evitar transfusões sanguíneas desnecessárias, devido a uma série de possíveis efeitos adversos. Além disso, muitos estudos encontraram associação estreita entre transfusão de concentrado de hemácias e ECN em prematuros. Por outro lado, a anemia também pode trazer uma séria de complicações ao RN envolvendo possível inadequada oxigenação tecidual como taquicardia, aumento das necessidades de oxigênio suplementar, aumento de episódios de apneia e dificuldade de ganho ponderal.

A hemácia é célula carreadora de O2 e diretamente ligada à oferta de oxigênio tecidual. Com este racional, é correto concluir que situações onde há alteração significativa no valor do hematócrito estarão diretamente relacionadas às alterações nos valores de rSO2.

Diversos autores têm indagado o possível papel que a aferição da rSO2 pode ocupar na indicação de transfusão sanguínea. Tentando definir o uso do NIRS como preditor de transfusão, seguem abaixo achados relacionados à transfusão:[67]

- A rScO2 estará em risco quando níveis de hemoglobina diminuírem de 9,7g/dl.
- Um significante aumento na rScO2 e diminuição nos sintomas de anemia foi descrito em crianças transfundidas com rScO2 menor que 55%.
- Em estudo piloto randomizado, RN pré-termos, com base em um protocolo convencional, receberam transfusão antes e com Hb mais alta, comparados com RN transfundidos com protocolo baseado no NIRS.
- Em uma série de casos, transfusões de concentrado de hemácias seguidas de diagnóstico de ECN, tiveram uma diminuição e maior variabilidade da rSmO2 comparadas com aqueles que não tiveram diagnóstico de ECN.

Os resultados dos estudos acima mencionados, por serem relacionados a estudos pequenos, não randomizados, de centros únicos e muitas vezes caráter piloto, merecem ser validados por estudos maiores e preferencialmente multicêntricos com protocolos bem definidos antes de serem aplicados em larga escala na prática clínica.

Indicações de Monitoramento (Monitoring indications)

Seguindo as evidências de aplicabilidade clínica descritas acima, encontramos as principais indicações de monitorização com NIRS na tabela 3. Ressalta-se que, sempre que disponível, utilizar simultaneamente os dois sítios de monitorização, cerebral e somático, acrescenta informações ao raciocínio clínico e pode auxiliar de forma importante no diagnóstico precoce de diversos acometimentos em pacientes de alto risco de lesão cerebral.

Tabela 3. Principais Indicações de Monitoramento com NIRS.

	**Indicações de Monitoramento**
**1**	EHI moderada/grave
**2**	Acidente vascular cerebral
**3**	Prematuridade extrema com complicações
**4**	Instabilidade hemodinâmica ou ventilatória
**5**	Cardiopatia Congênita Complexa
**6**	Hérnia Diafragmática
**7**	PCA com repercussão hemodinâmica
**8**	Anemia
**9**	ECMO

Valores de Referência (Reference Values)

O valor de normalidade é considerado de 55 a 85%, sendo essa uma faixa segura para os recém-nascidos.[82] Além do valor absoluto, deve-se atentar para a tendência do NIRS ao longo das

horas com especial atenção em caso de oscilação de mais de 15% mesmo estando dentro da faixa de segurança. A variação dos valores de saturação regional ao longo do tempo, principalmente correlacionada as mudanças de condutas, intercorrências clínicas e administração de medicações são de grande utilidade na prática clínica.

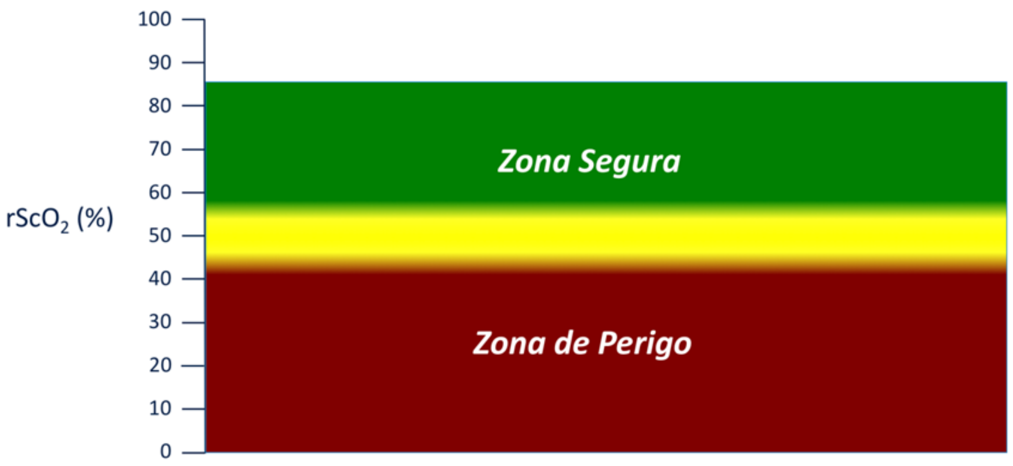

Figura 12. Zonas Alvo dos valores de referência de rSO2. Fonte: Alderliesten T, Dix L, Baerts W, Caicedo A, van Huffel S, Naulaers G, et al. Reference values of regional cerebral oxygen saturation during the first 3 days of life in preterm neonates. Pediatr Res. 2016; 79(1-1): 55-64. Dix LM, van Bel F, Baerts W, Lemmers PM. Comparing near-infrared spectroscopy devices and their sensors for monitoring regional cerebral oxygen saturation in the neonate. Pediatr Res. 2013; 74(5): 557-63. Adaptado.

Interpretação dos Valores do NIRS (NIRS values interpretation)

Diversos fatores podem alterar rSO2 em recém-nascidos, portanto em caso de valores anormais, deve-se raciocinar em torno do balanço entre a oferta e a demanda de oxigênio regional na tentativa de identificar a causa da alteração.

a) *Fatores que afetam a oferta de oxigênio*

- Concentração de Hemoglobina
- Saturação de Hemoglobina
- Débito Cardíaco (frequência cardíaca, pré-carga, contratilidade, pós-carga)
- Hipocapnia ou Hipercapnia

b) *Fatores que afetam a demanda de oxigênio*

- Aumentam a demanda:

- Febre
- Tremores
- Infecção
- Convulsão
- Dor

- Diminuem a demanda:

- Hipotermia
- Sedação
- Paralisia

Sempre que a rSO2 sair da zona alvo e/ou da tendência de forma importante, deve-se checar cada item descrito de acordo com o diagnóstico do paciente, para identificar a causa e planejar a melhor ação terapêutica. O resumo das principais ações está descrito na figura 13.

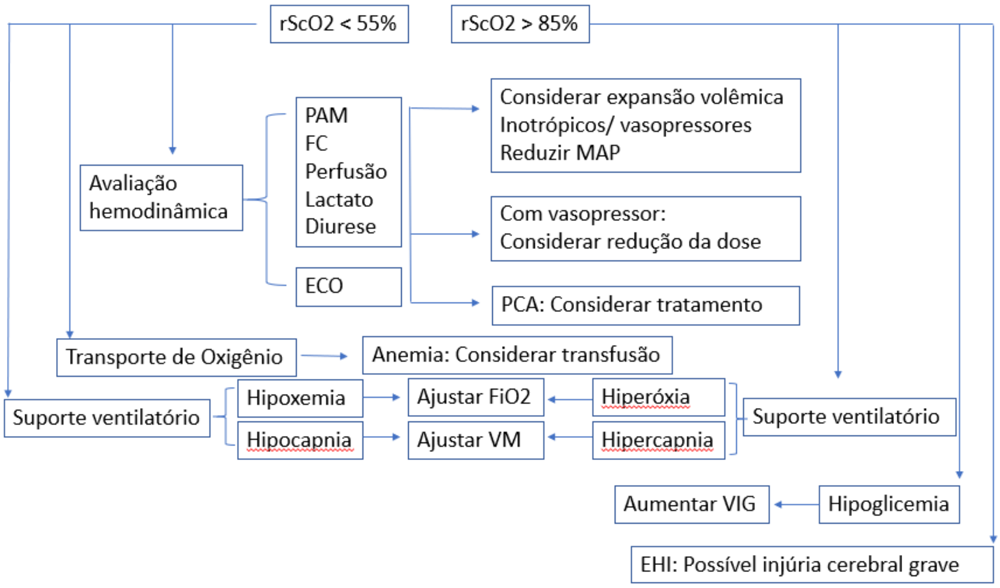

Figura 13. Fluxograma que resume as principais avaliações e ações frente a alteração no valor da rScO2.

CONCLUSÃO (Conclusion)

O conceito de UTI Neonatal Neurológica vem ganhando força nos últimos anos e inclui o cuidado focado na prevenção de sequelas neurológicas a curto e longo prazo. Com o uso de metodologias avançadas como o monitoramento cerebral contínuo com vídeo aEEG/EEG e NIRS espera-se antecipar diagnósticos e prover terapêutica precoce e eficaz na redução de injúria cerebral em recém-nascidos de alto risco.

REFERÊNCIAS BIBLIOGRÁFICAS (references)

1. Kurinczuk JJ, White-Koning M, Badawi N. Epidemiology of neonatal encephalopathy and hypoxic-ischaemic encephalopathy. Early Hum Dev. 2010;86(6):329-38.
2. World health report 2005: Make every mother and child count Geneva: WHO; 2005.
3. Lawn JE, Cousens S, Zupan J. 4 million neonatal deaths: When? Where? Why? Lancet. 2005;365(9462):891-900.
4. Lawn JE, Cousens SN, Wilczynska K. Estimating the causes of four million neonatal deaths in the year 2000: statistical annex. In: The world health report 2005 Geneva: WHO; 2005.
5. Shankaran S, Woldt E, Koepke T, Bedard MP, Nandyal N. Acute neonatal morbidity and long-term central nervous system sequelae of perinatal asphyxia in term infants. Early Hum Dev. 1991;25(2):135-48.
6. Robertson CMT. Long-term follow-up of term infants with perinatal asphyxia. In: Stevenson DK, Benitz WE, Sunshine P, editors. Fetal and neonatal brain injury. 3rd ed Cambridge University; New York: 2003. pp. 829-58.
7. De Vries LS, Jongmans MJ. Long-term outcome after neonatal hypoxic-ischemic encephalopathy. Arch Dis Child Fetal Neonatal Ed. 2010;95(3):F220-4.
8. Marlow N, Rose AS, Rands CE, Draper R. Neuropsychological and educational problems at school age associated with neonatal encephalopathy. Arch Dis Child Fetal Neonatal Ed. 2005;90(5):F380-7.
9. Blencowe H, Lee ACC, Cousens S, Bahalim A, Narwal R, Zhong N, et al. Preterm birth-associated neurodevelopmental impairment estimates at regional and global levels for 2010. Pediatr Res. 2013;74(Suppl 1):17-34.
10. Honeycutt A, Dunlap L, Chen H, al Homsi G. Economic Costs Associated with Mental Retardation, Cerebral Palsy, Hearing Loss, and Vision Impairment---United States, 2003. MMWR Morb Mortal Wkly Rep. 2004;53(3):57-9.
11. Boletim BPC – Benefício da Prestação Continuada. Ministério do Desenvolvimento Social e Combate à Fome, Secretaria Nacional da Assistência social, Ano 2015.

12. Van Meurs KP, Yan ES, Randall KS, Chock VY, Davis AS, Glennon CD, et al. Development of a NeuroNICU with a Broader Focus on All Newborns at Risk of Brain Injury: The First 2 Years. Am J Perinatol. 2018;35(12):1197-205.
13. Gluckman PD, Wyatt J, Azzopardi DV, et al. Selective head cooling with mild systemic hypothermia after neonatal encephalopathy: multicenter randomized trial. Lancet. 2005;365(9460):663-70.
14. Zhou WH, Cheng GQ, Shao XM, et al. Selective head cooling with mild systemic hypothermia after neonatal hypoxic-ischemic encephalopathy: a multicenter randomized controlled trial in China. J Pediatr. 2010;157(3):367-72.
15. Shankaran S, Laptook AR, Ehrenkranz RA, Tyson JE, McDonald SA, Donovan EF, et al. Whole-body hypothermia for neonates with hypoxic-ischemic encephalopathy. N Engl J Med. 2005;353(15):1574-84.
16. Azzopardi DV, Strohm B, Edwards AD, Dyet L, Halliday HL, Juszczak E, et al. Moderate hypothermia to treat perinatal asphyxial encephalopathy. N Engl J Med. 2009;361:1349-58.
17. Simbruner G, Mittal RA, Rohlmann F, Muche R, neo.nEURO.network Trial Participants. Systemic hypothermia after neonatal encephalopathy: outcomes of neo.nEURO.network RCT. Pediatrics. 2010;126(4):e771-8.
18. Jacobs SE, Morley CJ, Inder TE, Stetwart MJ, Smith KT, McNamara PJ, et al. Whole-body hypothermia for term and near-term newborns with hypoxic-ischemic encephalopathy: a randomized controlled trial. Arch PediatrAdolesc Med. 2011;165(8):692-700.
19. Tagin MA, Woolcott CG, Vincer MJ, Whyte RK, Stinson DA. Hypothermia for neonatal hypoxic ischemic encephalopathy: an updated systematic review and meta-analysis. Arch Pediatr Adolesc Med. 2012;166(6):558-66.
20. Edwards AD, Brocklehurst P, Gunn AJ, Halliday D, Juszczak E, Levene M, et al. Neurological outcomes at 18 months of age after moderate hypothermia for perinatal hypoxic ischemic encephalopathy: synthesis and meta-analysis of trial data. BMJ. 2010;9:340–c363.
21. Procianoy RS, Corso Al, Schoenardie BO, de Oliveira GPF, Longo MG, Silveira RC. Outcome and Feasibility after Seven Years of Therapeutic Hypothermia in Southern Brazil. Am J Perinatol. 2020;37(9):955-61.
22. Magalhães M, Rodrigues FPM, Chopard MRT, Melo VCA, Melhado A, Oliveira I, et al. Neuroprotective body hypothermia among newborns with hypoxic ischemic encephalopathy: three-year experience in a tertiary university hospital. A retrospective observational study. Sao Paulo Med J. 2015;133(4):314-9.
23. Procianoy RS. Hipotermia terapêutica. SBP. Departamento de Neonatologia. Documento científico. Disponível em: http://www.sbp.com.br/pdfs/hipotermia-terapeutica.pdf
24. Austin T, Ganado CC, Clarke P, O'Hare S, Merchant N, Vakharia B, et al. Clinical Guideline: Guidelines for Management of Infants with Suspected Hypoxic Ischaemic Encephalopathy (HIE). 2016.
25. Regier DA, Petrou S, Henderson J, Eddama O, Patel N, Strohm B, et al. Cost-effectiveness of therapeutic hypothermia to treat neonatal encephalopathy. Value in Health. 2020;13(6):695-702.
26. Ministério da Saúde. DATASUS. Disponível em: http://tabnet.datasus.gov.br/cgi/tabcgi.exe?sinasc/cnv/nvuf.def.
27. Variane GF, Cunha LM, Pinto P, Brandao P, Mascaretti RS, Magalhães M, et al. Therapeutic Hypothermia in Brazil: A MultiProfessional National Survey. Am J Perinatol. 2019;36(11):1150-1156.
28. Schettler KF. The aEEG Booklet A quick overview for the practical routine. Nihon Kohden, 2012.
29. Variane GFT, Magalhães M, Gasperine R, Alves HCBR, Scoppetta TLPD, Figueredo RJG, et al. Early amplitude-integrated electroencephalography for monitoring neonates at high risk. J Pediatr. 2017;93(5):460-6.
30. Rakshasbhuvankar A, Paul S, Nagarajan L, Ghosh S, Rao S. Amplitude-integrated EEG for detection of neonatal seizures: a systematic review. Seizure. 2015;33:90-8.
31. Shah DK, Mackay MT, Lavery S, Watson S, Harvey AS, Zempel J, et al. Accuracy of bedside electroencephalographic monitoring in comparison with simultaneous continuous conventional

electroencephalography for detection of neonatal seizures in term infants. Pediatrics. 2008;121(6):1146-54.
32. Toet MC, van der Meij W, de Vries LS, Uiterwaal CSPM, van Huffelen KC. Comparison between simultaneously recorded amplitude integrated electroencephalogram (cerebral function monitor) and standard electroencephalogram in neonates. Pediatrics, 2002;109(5):772-9.
33. Glass HC, Wusthoff CJ, Shellhaas RA. Amplitude Integrated EEG: The Child Neurologist's Perspective. J Child Neurol. 2013;28(10):1342-50.
34. Chandrasekaran M, Chaban B, Montaldo P, Thayyil S. Predictive value of amplitude-integrated EEG (aEEG) after rescue hypothermia neuroprotection for hypoxic ischemic encephalopathy: a meta-analysis. J Perinatol. 2017;37(6):684-9.
35. Klebermass K, Olischar M, Waldhoer T, Fuiko R, Pollak A, Weninger M. Amplitude-integrated EEG pattern predicts further outcome in preterm infants. Pediatr Res. 2011;70(1):102-8.
36. Latal B, Wohlrab G, Brotschi B, Beck I, Knirsh I, Bernet V. Postoperative amplitude-integrated electroencephalography predicts four-year neurodevelopmental outcome in children with complex congenital heart disease. J Pediatr. 2016;178:55-60.
37. Hellström-Westas L, Rosén I, de Vries LS, Greisen G. Amplitude-integrated EEG Classification and Interpretation in Preterm and Term Infants. NeoReviews. 2006;7(2):e76-e87.
38. Toso PA, González AJ, Pérez ME, Kattan J, Fabres JG, Tapia JL, et al. Clinical utility of early amplitude integrated EEG in monitoring term newborns at risk of neurological injury. J Pediatr (Rio J). 2014;90(2):143-8.
39. Marinho TF. Recomendação da SBNC para localização de eletrodos e montagens de EEG. [Internet] São Paulo: Sociedade Brasileira de Neurofisiologia Clínica; 2017 [Acessado 16 dez 2016]. Disponível em: https://sbnc.org.br/wp-content/uploads/2015/05/1512584738_Norma_montagens_EEG_.pdf
40. Wusthoff CJ. Diagnosing Neonatal Seizures and Status Epilepticus. J Clin Neurophysiol 2013;30(2):115-21.
41. Volpe JJ. Neonatal seizures. Philadelphia: WB Saunders; 1995.
42. Murray DM, Boylan GB, Ali I, Ryan CA, Murphy BP, Connolly S. Defining the gap between electrographic seizure burden, clinical expression and staff recognition of neonatal seizures. Arch Dis Child Fetal Neonatal Ed. 2008;93(3):F187-91.
43. Abend NS, Wusthoff CJ, Goldberg EM, Dlugos DJ. Electrographic seizures and status epilepticus in critically ill children and neonates with encephalopathy. Lancet Neurol. 2013;12(12):1170-9.
44. van Rooij LG, Toet MC, van Huffelen AC, Groenendaal F, Laan W, Zecic A, et al Effect of treatment of subclinical neonatal seizures detected with aEEG: randomized, controlled trial. Pediatrics. 2010;125(2):e358-66.
45. Srinivasakumar P, Zempel J, Trivedi S, Wallendorf M, Rao R, Smith B, et al. Treating EEG Seizures in Hypoxic Ischemic Encephalopathy: A Randomized Controlled Trial. Pediatrics. 2015;136(5):e1302-9.
46. Bashir RA, Espinoza L, Vayalthrikkovil S, Buchhalter J, Irvine L, Bello-Espinosa L, et al. EImplementation of a Neurocritical Care Program: Improved Seizure Detection and Decreased Antiseizure Medication at Discharge in Neonates with Hypoxic-Ischemic Encephalopathy. Pediatr Neurol. 2016;64:38-43.
47. Jan S, Northington FJ, Parkinson CM, Stafstrom CE. EEG Monitoring Technique Influences the Management of Hypoxic-Ischemic Seizures in Neonates Undergoing Therapeutic Hypothermia. Dev Neurosci. 2017;39(1-4):82-8.
48. Del Rio R, Ochoa C, Alarcon A, Arnáez J, Blanco D, García-Alix A. Amplitude Integrated Electroencephalogram as a Prognostic Tool in Neonates with Hypoxic-Ischemic Encephalopathy: A Systematic Review. PLoS One. 2016;11(11):e0165744.
49. Hellström-Westas L, Rosén I, Svenningsen NW. Predictive value of early continuous amplitude integrated EEG recordings on outcome after severe birth asphyxia in full term infants. Arch Dis Child Fetal Neonatal Ed. 1995;72(1):F34-8.
50. Skranes JH, Løhaugen G, Schumacher EM, Osredkar D, Server A, Cowan FM, et al. Amplitude-Integrated Electroencephalography Improves the Identification of Infants with

Encephalopathy for Therapeutic Hypothermia and Predicts Neurodevelopmental Outcomes at 2 Years of Age. JPediatr. 2017;187:34-42.
51. Thoresen M, Hellström-Westas L, Liu X, de Vries LS. Effect of hypothermia on amplitude-integrated electroencephalogram in infants with asphyxia. Pediatrics. 2010;126:e131–9.
52. Wusthoff CJ, Dlugos DJ, Gutierrez-Colina A, Wang A, Cook N, Donnelly M, et al. Electrographic seizures during therapeutic hypothermia for neonatal hypoxic-ischemic encephalopathy. J Child Neurol. 2011;26(6):724-8.
53. Glass HC, Glidden D, Jeremy RJ, Barkovich AJ, Ferriero DM, Miller SP. Clinical neonatal seizures are independently associated with outcome in infants at risk for hypoxic–ischemic brain injury. J Pediatr. 2009;155(3):318-23.
54. Tekgul H, Gauvreau K, Soul J, Murphy L, Robertson R, Stewart J, et al. The current etiologic profile and neurodevelopmental outcome of seizures in term newborn infants. Pediatrics 2006;117(4):1270-80.
55. Hellström-Westas L, Rosén I, Svenningsen NW. Cerebral function monitoring during the first week of life in extremely small low birthweight (ESLBW) infants. Neuropediatrics. 1991;22(1):27-32.
56. Shah DK, Zempel J, Barton T, Lukas K, Inder TE. Electrographic seizures in preterm infants during the first week of life are associated with cerebral injury. Pediatr Res. 2010;67(1):102-6.
57. Davis AS, Hintz SR, Van Meurs KP, Li L, Das A, Stoll BJ, et al. Seizures in extremely low birth weight infants are associated with adverse outcome. J Pediatr. 2010;157(5):720-5.
58. Vesoulis ZA, Inder TE, Woodward LJ, Buse B, Vavasseur, Mathur AM. Early electrographic seizures, brain injury and neurodevelopmental risk in the very preterm infant. Pediatr Res. 2014;75(4):564-9.
59. Gunn JK, Beca J, Hunt RW, Olischer M, Shrkerdemian LS. Perioperative amplitude-integrated EEG and neurodevelopment in infants with congenital heart disease. Intensive Care Med. 2012;38(9):1539-47.
60. Latal B, Wohlrab G, Brotschi B, Beck I, Knirsch W, Bernet V. Postoperative amplitude-integrated electroencephalography predicts four-year neurodevelopmental outcome in children with complex congenital heart disease. J Pediatr. 2016;178:55-60.
61. Olischar M, Shany E, Aygün C, Azzopardi D, Hunt RW, Toet MC, et al. Amplitude-integrated electroencephalography in newborns with inborn errors of metabolism. Neonatology. 2012;102(3):203-11.
62. Helderman JB, Welch CD, Leng X, O`Shea TM. Sepsis-associated electroencephalographic changes in extremely low gestational age neonates. Early Hum Dev. 2010;86(8):509-13.
63. Hellström-Westas L, Bell AH, Skov L, Greisen G, Svenningsen NW. Cerebral depression following surfactant treatment in preterm neonates. Pediatrics. 1992;89(4 Pt 1):643-7.
64. Lima A, Bakker J. Espectroscopia no infravermelho próximo para a monitorização da perfusão tecidual. Ver Bras Ter Intensiva. 2011;23(3):341-51.
65. Engelhardt B, Gillam-Krakauer M. Use of Near-Infrared Spectroscopy in the Management of Patients in Neonatal Intensive Care Units – An Example of Implementation of a New Technology, Infrared Spectroscopy-Life and Biomedical Sciences. In Tech; 2012.
66. Somanetics Corporation. INVOS® Cerebral/Somatic Oximeter Quick Reference Guide for Pediatric Use. Troy, Michigan, USA.
67. Sood BG, McLaughlin K, Cortez J. Near-infrared spectroscopy: applications in neonates. Semin Fetal Neonatal Med. 2015;20(3):164-72.
68. Alderliesten T, Dix L, Baerts W, Caicedo A, van Huffel S, Naulaers G, et al. Reference values of regional cerebral oxygen saturation during the first 3 days of life in preterm neonates. Pediatr Res. 2016;79(1-1):55-64.
69. Chock VY, Variane GFT, Netto A, Van Meurs KP. NIRS improves hemodynamic monitoring and detection of risk for cerebral injury: cases in the neonatal intensive care nursery. J Matern Fetal Neonatal Med. 2018;29: 1-9.
70. Martini S, Corvaglia L. Splanchnic NIRS monitoring in neonatal care: rationale, current applications and future perspectives. J Perinatol. 2018;38(5):431-43.

71. Jain SV, Pagano L, Gillam-Krakauer M, Slaughter JC, Pruthi S, Engelhardt B. Cerebral regional oxygen saturation trends in infants with hypoxic-ischemic encephalopathy. Early Hum Dev. 2017;113:55–61.
72. Lemmers PM, Zwanenburg RJ, Benders MJ, de Vries LS, Groenendaal F, van Bel F, et al. Cerebral oxygenation and brain activity after perinatal asphyxia: does hypothermia change their prognostic value? Pediatr Res. 2013;74(2):180-5.
73. Hyttel-Sorensen S, Pellicer A, Alderliesten T, Austin T, van Bel F, Benders M, et al. Cerebral near infrared spectroscopy oximetry in extremely preterm infants: phase II randomised clinical trial. BMJ. 2015;350:g7635.
74. Vesoulis ZA, Lust CE, Liao SM, Trivedi SB, Mathur AM. Early hyperoxia burden detected by cerebral near-infrared spectroscopy is superior to pulse oximetry for prediction of severe retinopathy of prematurity. J Perinatol. 2016;36(11):966-71.
75. Chock VY, Rose LA, Mante JV, Punn R. Near-infrared spectroscopy for detection of a significant patent ductus arteriosus. Pediatr Res. 2016;80(5):675-80.
76. Dodge-Khatami J, Gottschalk U, Eulenburg C, Wendt U, Schnegg C, Rebel M, et al. Prognostic value of perioperative near-infrared spectroscopy during neonatal and infant congenital heart surgery for adverse in-hospital clinical events. World J Pediatr Congenit Heart Surg. 2012;3(2):221-8.
77. Colasacco C, Worthen M, Peterson B, Lamberti J, Spear R. Near-Infrared Spectroscopy Monitoring to Predict Postoperative Renal Insufficiency Following repair of Congenital Heart Disease. World J Pediatr Congenit Heart Surg. 2011;2(4):536-40.
78. Hoffman GM, Guanayem NS, Scott JP, Tweddell JS, Mitchell ME, Mussatto KA. Postoperative Cerebral and Somatic Near-Infrared Spectroscopy Saturations and Outcome in Hypoplastic Left Heart Syndrome. Ann Thorac Surg. 2017;103(5):1527-35.
79. Clair MP, Rambaud J, Flahault A, Guedj R, Guilbert J, Guellec I, et al. Prognostic value of cerebral tissue oxygen saturation during neonatal extracorporeal membrane oxygenation. PLoS One. 2017;12(3):e0172991.
80. Li T, Duan M, Li K, Yu G, Ruan Z. Bedside monitoring of patients with shock using a portable spatially-resolved near-infrared spectroscopy. Biomed Opt Express. 2015;6(9):3431-6.
81. Alderliesten T, Lemmers P, van Haastert IC, de Vries LS, Bonestroo HJ, Baerts W, et al. Hypotension in preterm neonates: low blood pressure alone does not affect neurodevelopmental outcome. J Pediatr. 2014;164(5):986-91.
82. Jeon GW. Clinical Application of Near-Infrared Spectroscopy in Neonates. Neonatal Med. 2019;26(3):121-7.

ANEXO II (Apendix II)

PROTOCOLO DE HIPOTERMIA TERAPÊUTICA PARA RECÉM-NASCIDOS COM ENCEFALOPATIA HIPÓXICO-ISQUÊMICA
(Therapeutic Hypothermia Protocol)
Equipe PBSF-Protecting Brains & Saving Futures

A neonatologia é uma das especialidades médicas que mais evoluíram nas últimas décadas, com importante redução da mortalidade neonatal. Entretanto, por diversas vezes esse incremento da sobrevida não foi acompanhado de uma proteção ao neurodesenvolvimento adequado.
Algumas doenças no período neonatal que estão associadas a alto risco de desenvolvimento de sequelas. A asfixia perinatal, doença com incidência de 1 a 8 por 1.000 nascidos vivos a termo representa a terceira causa mais comum de morte neonatal (23%) após nascimento prematuro (28%) e infecções graves (26%). Apesar dos importantes avanços citados nos cuidados perinatais nas últimas décadas, a asfixia continua sendo uma afecção grave, que pode levar a condição denominada encefalopatia hipóxico-isquêmica (EIH).

Os recém-nascidos com encefalopatia grave têm alto risco de morte, paralisia cerebral e retardo mental entre os sobreviventes. Os recém-nascidos com encefalopatia moderada apresentam déficits motores significativos, deficiência motora fina, comprometimento da memória, disfunção visual, aumento da hiperatividade e atraso na prontidão escolar.

A fisiopatologia da injúria cerebral secundária a EHI está relacionada a duas fases, a falha energética primária e secundária. A falha energética primária é caracterizada por redução no fluxo sanguíneo e oxigenação cerebral, com consequente acidose tecidual. Esta primeira fase está associada a uma cascata excitotóxica-oxidativa, com excessiva estimulação de receptores de neurotransmissores e despolarização de membrana, levando ao aumento de cálcio intracelular e desregulação osmótica. O cálcio intracelular leva a liberação de óxido nítrico neuronal, o que pode interromper a respiração mitocondrial, resultando em apoptose neuronal. A falha energética secundária envolve a continuação do processo da cascata excitotóxica-oxidativa, apoptose, inflamação, alteração dos fatores de crescimento e de síntese protéica. O intervalo entre a falha primária e secundária de energia é representada pela fase latente, com duração aproximada de 6 horas, o que corresponde a janela terapêutica.

Estudos randomizados e controlados demonstram que a hipotermia terapêutica para recém-nascidos com EHI moderada ou grave é um tratamento seguro e efetivo, com redução da mortalidade e sequelas neurológicas a longo prazo, sendo a terapia neuroprotetora padrão ouro neste grupo de pacientes.

Todo recém-nascido com EHI deve ser avaliado quanto à elegibilidade em receber o resfriamento corpóreo. Os critérios de inclusão e exclusão listados abaixo devem ser revisados quando avaliamos esses recém-nascidos. **O resfriamento deve ser iniciado nas primeiras 6 horas após o nascimento.**

1) **Seleção dos pacientes na sala de parto elegíveis para avaliação de Hipotermia Terapêutica: (therapeutic hypothermia - patient selection in the delivery room)**

Recém-nascidos com idade gestacional maior ou igual a 35 semanas e peso de nascimento maior ou igual a 1.800 gramas, sem malformação congênita maior que apresente os itens abaixo:

- Necessidade de reanimação neonatal em sala de parto e;
- Suspeita de Asfixia com APGAR menor ou igual a 7 no 5° minuto de vida e/ou presença de evento perinatal agudo*.

*Evento Perinatal Agudo:
- Desacelerações Tardias (DIP II)
- Prolapso ou rotura de cordão umbilical
- Rotura Uterina
- Hemorragias do 3° trimestre

- Parada Cardiorrespiratória da mãe
- Outras situações de sofrimento fetal agudo

2) Se paciente elegível para avaliação de Hipotermia Terapêutica: (if eligle for TH)

- Após estabilização em Sala de Parto, coletar gasometria do cordão umbilical ou do próprio RN dentro da primeira hora de vida;
- Reanimação e Transporte a UTI Neonatal de acordo com as diretrizes da Sociedade Brasileira de Pediatria;
- Controle térmico na Sala de Parto e na admissão em UTI Neonatal. <u>Evitar hipertermia.</u> Estudos indicam que a presença de hipertermia tem relação com aumento do risco de morte ou sequelas neurológicas nos pacientes asfixiados.

3) Na admissão em UTI Neonatal (at NICU admission)

A avaliação para indicação de Hipotermia Terapêutica inclui gasometria arterial na primeira hora de vida, avaliação de encefalopatia clínica através da escala de avaliação neurológica de SARNAT e SARNAT modificado, e se disponível, o uso do monitoramento cerebral com eletroencefalograma de amplitude integrada.

- Se Gasometria de cordão ou arterial na primeira hora de vida com pH menor ou igual a 7,0 e/ou BE menor ou igual a -16, o próximo passo será avaliar presença de encefalopatia hipóxico-isquêmica;
- Se não houver coleta de gasometria na primeira hora de vida (transferência externa) ou se a gasometria arterial na primeira hora de vida apresentar pH entre 7,01 e 7,15 e/ou BE entre -10 e -15,9, avaliar os fatores abaixo:

- Presença de evento Perinatal Agudo associado a;
- APGAR menor ou igual a 5 no 10° minuto de vida e/ou;
- Necessidade de Suporte Ventilatório por mais de 10 minutos de vida;

→ Avaliar presença de Encefalopatia Hipóxico-Isquêmica

Avaliação dos critérios para EHI: (criteria for HIE)

A avaliação de EHI deve ser realizada através da escala de SARNAT e SARNAT modificado e uso do Vídeo aEEG/EEG, se disponível.

- Se presença de 3 ou mais critérios clínicos de EHI moderada ou grave (SARNAT) e/ou;
- Presença de Crise Convulsiva Clínica e/ou;
- aEEG com depressão da atividade elétrica cerebral de base (Amplitude Mínima abaixo de 5 µV) e/ou presença de atividade epiléptica.

→ Iniciar Hipotermia Terapêutica nas primeiras 6 horas de vida, se não houver critérios de exclusão.

Critérios de exclusão: (exclusion criteria)

- Idade gestacional abaixo de 35 semanas.
- Peso de nascimento menor que 1.800 g.
- Malformações congênitas graves e/ou incompatíveis com a vida.
- Sangramento ativo muito importante sem controle com todo arsenal terapêutico disponível

(Vitamina K, plasma, crioprecipitado e plaquetas).

- Hipertensão pulmonar não controlada e com hipoxemia refratária mesmo após utilizar todo arsenal terapêutico disponível, incluindo óxido nítrico, drogas vasoativas e ventilação mecânica adequada.
- Choque refratário sem controle com toda terapêutica disponível ou bradicardia persistente (FC <60bpm) que não melhore com uso de atropina.

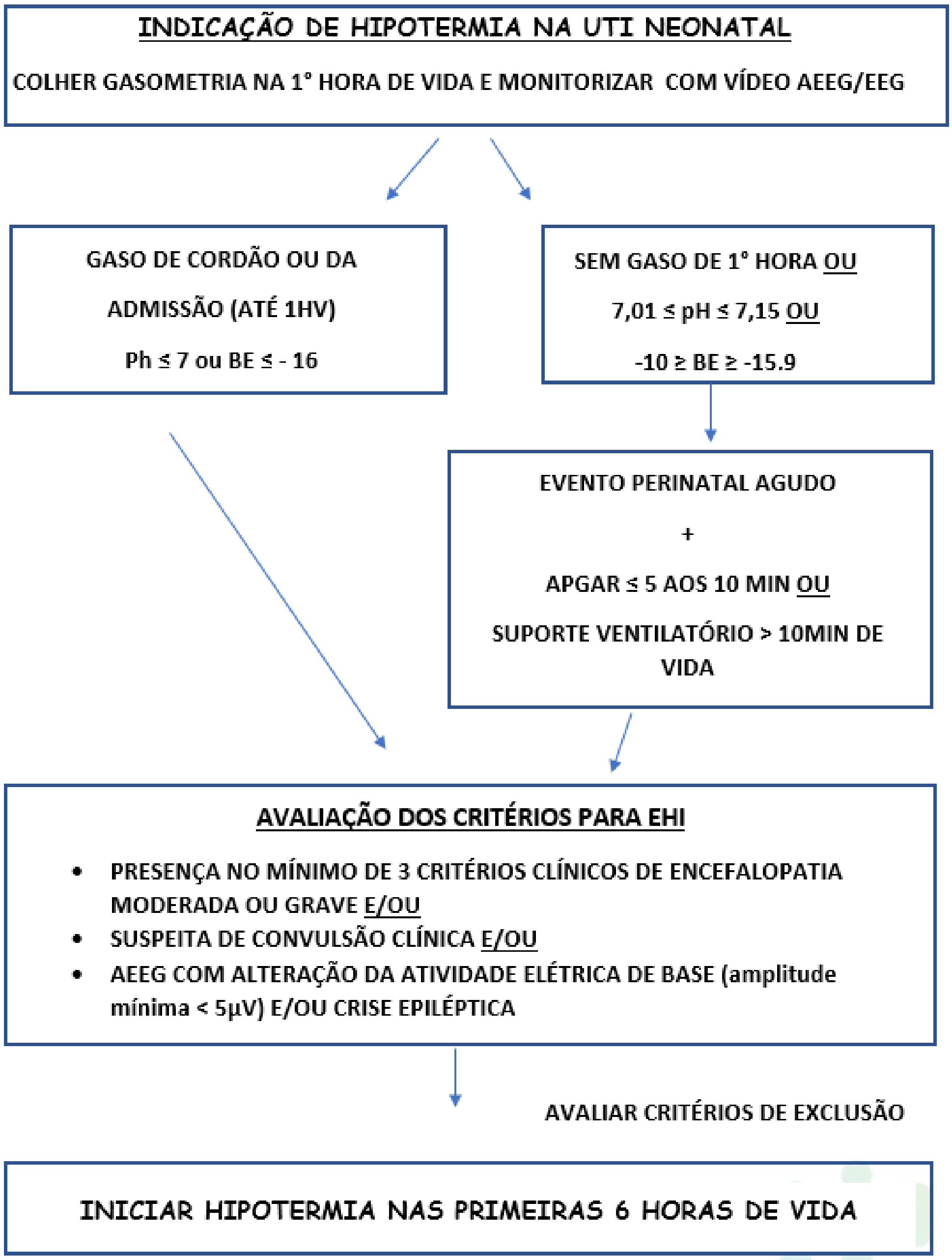

Figura 1. Fluxograma para Indicação de Hipotermia Terapêutica.

Categoria	Normal	EHI Leve	EHI Moderada	EHI Grave
1) Nível de Consciência	Alerta, responsivo	Hiperalerta, responde à mínimos estímulos	Letárgico	Estupor ou Coma
2) Atividade espontânea	Espontânea	Espontânea ou diminuída	Diminuída	Sem atividade
3) Postura	Normal	Leve flexão distal (punho e dedos)	Flexão distal ou extensão completa	Descerebração
4) Tônus	Em flexão	Em flexão	Hipotonia (focal ou geral) ou hipertonia	Flácido ou rígido
5) Reflexos Primitivos				
Sucção	Forte	Fraca	Fraca ou mordida	Ausente
Moro	Completo	Normal ou Incompleto	Incompleto	Ausente
6) Sistema Autonômico				
Pupilas	Fotorreagentes	Midríase Leve	Miose	Arreativas
Frequência Cardíaca	100 a 160 bpm	Taquicardia	Bradicardia	Variável
Respiração	Regular	Taquipnéia	Periódica	Apnéia ou Ventilação Mecânica

Figura 2. Escala de SARNAT e SARNAT modificado por Levene e Volpe.

Visão Geral (General Guidelines)

1. **Início da hipotermia:** Até 6 horas de vida;

2. **Duração da hipotermia:** 72 horas;

3. **Método de resfriamento:** Resfriamento corporal total;

4. **Dispositivos de resfriamento:** A metodologia ideal para o resfriamento consiste no uso de colchões/pads térmicos servo-controlados que regulam automaticamente a temperatura para o alvo. Se indisponível, considerar uso de bolsas de gelo, desde que ocorra ajuste rígido de acordo com aferição por monitorização contínua de temperatura;

5. **Controle da temperatura:** Monitorização contínua da temperatura retal ou esofágica, com anotações do controle térmico a cada 30 minutos nas primeiras 6 horas e após de hora em hora;

6. **Temperatura alvo retal/esofágica:** entre 33 º C a 34ºC (33,5º C);

7. **Acesso vascular:** Cateterismo umbilical arterial e venoso;

8. **Dieta:** Jejum durante período de resfriamento e até o final do reaquecimento. Considerar colostroterapia;

9. **Nutrição parenteral:** Utilizar após 24 horas de vida, se estabilidade hemodinâmica;

10. **Oferta hídrica:** Preconiza-se restrição hídrica, iniciando oferta com 50 a 60 ml/kg/dia e ajuste conforme balanço hídrico e peso;

11. **Suporte Ventilatório:** Indicação de suporte conforme a necessidade. Não é obrigatório estar intubado, sendo que grande parte dos pacientes permanecem estáveis em ar ambiente durante todo o protocolo de hipotermia. Em pacientes ventilados, atenção especial em evitar hipóxia, hiperóxia, hipocapnia ou hipercapnia.

12. **Sedação:** Iniciar fentanil ou morfina em doses baixas e titular conforme score de dor;

13. **Monitorização contínua cardíaca e de oximetria;**

14. **Controle de Pressão Arterial:** se disponível, controlar PA invasiva através de cateter arterial umbilical. Na indisponibilidade, realizar controle de PA não invasiva de hora em hora;

15. **Cateterismo vesical:** Manter até o final do reaquecimento, se diurese estável;

16. **Cuidados em UTI:** Manipulação Mínima e agrupamento dos cuidados pela equipe multidisciplinar;

17. **Cuidados com a pele:** O paciente pode ficar em posição supina ou prona, rodiziando essas posições, além de frequente exame da pele a cada 2 horas;

18. **Exames laboratoriais:** segue abaixo sugestão para coleta de exames laboratoriais, que deverão ser ajustados individualmente de acordo com cada caso;

**Cordão ou 1° Hora de Vida**	**1° Hora de Hipotermia**	**6 Horas de Hipotermia**	**24 Horas de Vida**	**48 Horas de Vida**	**72 Horas de Vida**	**Após 12 Horas Reaquecimento**
Gasometria	Gasometria	Gasometria	Gasometria	Gasometria	Gasometria	Gasometria

			Hemograma		Hemograma	
			Coagulograma		Coagulograma	
		Eletrólitos	Eletrólitos		Eletrólitos	Eletrólitos
			Função renal		Função renal	
		CPK, CKMB, DHL e troponina	CPK, CKMB, DHL e troponina			

19. **Ecocardiograma funcional:** Útil na avaliação hemodinâmica incluindo função miocárdica, volemia, débito cardíaco e avaliação de hipertensão pulmonar;

20. **Dispositivos de monitorização cerebral:** instalar vídeo aEEG/EEG e NIRS o mais precoce possível e manter até 24 horas após final do reaquecimento;

- Eletroencefalograma de Amplitude Integrada associado à leitura de EEG bruto: O período neonatal é o de maior incidência para crises convulsivas, sendo que a asfixia perinatal representa a principal causa de convulsão nos recém-nascidos a termo. Além da alta incidência, a maioria das crises epilépticas no período neonatal são subclínicas, e somente possíveis de diagnosticar durante monitorização eletroencefalográfica.

A monitorização com aEEG/EEG também permite avaliação de função cerebral em tempo real, e oferece a informação do tempo para traçado normal, que se relaciona a prognóstico neurológico.

- NIRS: Estudos indicam que a presença de saturação regional cerebral elevada após 24 horas de vida, se mantendo em valores acima de 90%, se relaciona com pior prognóstico. Isso ocorre por redução na taxa de extração de oxigênio secundária a lesão neuronal extensa.

21. **Avaliação neurológica:** utilizar escala de SARNAT modificada para avaliação clínica neurológica diariamente;

22. **USG Transfontanela:** se possível com doppler para avaliação do Índice de Resistência. Realizar no 1° dia de vida (útil para diagnóstico de sangramento grave com desvio de linha média que poderia contraindicar o resfriamento) e após o reaquecimento;

23. **Ressonância Magnética:** a realização do exame entre o 4° e 12° dia de vida pode trazer informações importantes quanto ao prognóstico tardio destes pacientes.

Possíveis Intercorrências (possible adverse effects)

- **Bradicardia sinusal:** atenção aos sinais de repercussão hemodinâmica. Na maioria dos casos é bem tolerada e não exige medicações adicionais.

Se FC < 60bpm, considerar atropina.

- **Oligúria:** pode ocorrer pela injúria renal.

- Considerar expansão volêmica somente se evidência de hipovolemia. Exemplo: sangramentos ou hipovolemia evidenciada em ecocardiograma funcional;
- Considerar uso de diurético e albumina conforme necessidade;
- Drogas vasoativas de acordo com parâmetros hemodinâmicos;
- Não retardar indicação de diálise em recém-nascidos oligoanúricos, sem resposta as medidas

anteriores.

- **Hipotensão arterial:**

- Avaliar outros parâmetros hemodinâmicos e considerar expansão volêmica e droga vasoativa (em especial inotrópicos) de acordo com cada caso;
- Realizar ecocardiograma funcional se disponível, para melhor avaliação.

- **Acidose Metabólica:** Após o nascimento, com suporte hemodinâmico e ventilatório adequado, ocorre melhora progressiva e espontânea da acidose metabólica. A administração de bicarbonato para correção da acidose não está recomendada de rotina.

- **Distúrbios hidroeletrolíticos:**

  - Hiponatremia é um achado frequente, sendo que a hiponatremia dilucional é a principal causa. Ajustar aporte hídrico e reposição conforme a necessidade.
  - Hipocalcemia: Atenção ao uso indiscriminado de cálcio pelo mecanismo de lesão neuronal relacionado ao influxo celular. Se for necessário reposição, evitar sobrecargas.

- **Sangramentos:**

- Considerar transfusão de plaquetas se abaixo de 50.000 e em especial quando relacionada a sangramento atibo;
- Avaliar necessidade de vitamina K, crioprecipitado, plasma de acordo com coagulograma e presença de sangramento ativo;

- **Hipertensão Pulmonar:**

- Tratamento de acordo com a necessidade e protocolo institucional, incluindo uso de drogas vasoativas e óxido nítrico, além de suporte ventilatório adequado;

- **Crises Convulsivas:**

- Tratar crises clínicas e subclínicas evidenciadas ao aEEG/EEG;
- Medicação de primeira escolha: fenobarbital;

- **Vias aéreas:** durante o período de hipotermia, as secreções em vias aéreas podem aumentar e tendem a se tornar mais espessas, tornando-se necessário atendimento fisioterápico regularmente.

- **Adiponecrose:** lesões raras caracterizadas por nódulos ou placas subcutâneas que ocorrem devido ao insulto hipóxico-isquêmico e podem ser detectadas logo ao nascimento ou até nas primeiras semanas de vida, com localização mais frequente em dorso, face, coxas e braços. A evolução tende a ser benigna e com resolução espontânea das lesões, porém podem estar associadas a alterações sistêmicas como hipoglicemia, anemia, plaquetopenia e hipercalcemia. Atenção aos sinais clínicos de hipercalcemia tardia e controle laboratorial de cálcio.

Período de Reaquecimento (Re-warming)

- Reaquecer lentamente, com elevação da temperatura entre 0,2 a 0,5º C por hora;

- Considerar suspender colchão térmico/sistema de controle de temperatura servo-controlado ao atingir temperatura retal entre 36,5 – 36,8ºC;

- Manter a monitorização de temperatura retal contínua até 24 horas após o reaquecimento;
- Evitar hipertermia;
- Período de maior risco para hipotensão arterial e crises convulsivas;
- Monitorização clínica rigorosa até 24 horas após final do reaquecimento.

Indicações de suspensão do Protocolo de Hipotermia Terapêutica (when to stop TH)

- Sangramento ativo muito importante sem controle com todo arsenal terapêutico disponível (Vitamina K, plasma, crioprecipitado e plaquetas).
- Hipertensão pulmonar não controlada (com hipoxemia refratária) mesmo com todo arsenal terapêutico disponível, incluindo óxido nítrico, drogas vasoativas e ventilação mecânica adequada.
- Choque refratário sem controle com toda terapêutica disponível ou bradicardia persistente (FC <60bpm) que não melhore com uso de atropina.
- **Atenção:** Na suspensão do protocolo, o reaquecimento deverá ser feito lentamente, respeitando a velocidade máxima de $0,5^{O}C$ por hora.

Referências Bibliográficas: (references)

1. Kurinczuk JJ, White-Koning M, Badawi N. Epidemiology of neonatal encephalopathy and hypoxic-ischemic encephalopathy. Early Hum Dev. 2010;86(6):329-38.
2. Lawn JE, Cousens S, Zupan J. 4 million neonatal deaths: When? Where? Why? Lancet. 2005;365(9462):891-900.
3. Lawn JE, Cousens SN, Wilczynska K. Estimating the causes of four million neonatal deaths in the year 2000: statistical annex. In: *The world health report 2005* Geneva: WHO; 2005.
4. De Vries LS, Jongmans MJ. Long-term outcome after neonatal hypoxic-ischemic encephalopathy. Arch Dis Child Fetal Neonatal Ed. 2010;95(3):F220-4.
5. Johnston MV, Fatemi A, Wilson MA, Northington F. Treatment advances in neonatal neuroprotection and neurointensive care. Lancet Neurol. 2011;10(4):372-82.
6. Cross JL, Meloni BP, Bakker AJ, Lee S. Modes of neuronal calcium entry and homeostasis following cerebral ischemia. Stroke Res Treat. 2010;(11):316862.
7. Shankaran S. Therapeutic Hypothermia for Neonatal Encephalopathy. Curr Treat Options Neurol. 2012;14(6):608-19.
8. Jacobs S, Hunt R, Tarnow-Mordi W, Inder T, Danis P. Cooling for newborns with hypoxic ischaemic encephalopathy. Cochrane Database Syst Rev. 2007;(4):CD003311.

9. Jacobs SE, Morley CJ, Inder TE, Stewart MJ, Smith KR, McNamara PJ, et al. Whole-body hypothermia for term and near-term newborns with hypoxic-ischemic encephalopathy: a randomized controlled trial. Arch Pediatr Adolesc Med. 2011;165(8):692-700.
10. Shankaran S, Laptook AR, Ehrenkranz RA, Tyson JE, McDonald SA, Donovan EF, et al. Whole-body hypothermia for neonates with hypoxic-ischemic encephalopathy. N Engl J Med. 2005;353(15):1574-84.
11. Tagin MA, Woolcott CG, Vincer MJ, Whyte RK, Atinson DA. Hypothermia for neonatal hypoxic ischemic encephalopathy: an updated systematic review and meta-analysis. Arch Pediatr Adolesc Med. 2012;166(6):558-66.
12. Laptook A, Tyson J, Shankaran S, McDonald S, Ehrenkranz R, Fanaroff A, et al. Elevated temperature after hypoxic-ischemic encephalopathy: A risk factor for adverse outcome. Pediatrics. 2008;122(3):491-9.
13. Thoresen M, Hellstrom-Westas L, Liu X, de Vries LS. Effect of hypothermia on amplitude-integrated electroencephalogram in infants with asphyxia. Pediatrics. 2010;126(1):e131-9.
14. Chandrasekaran M, Chaban B, Montaldo P, Thayyil S. Predictive value of amplitude-integrated EEG (aEEG) after rescue hypothermic neuroprotection for hypoxic ischemic encephalopathy: a meta-analysis. J Perinatol. 2017;37(6):684-9.
15. Azzopardi DV, Strohm B, Edwards AD, Dyet L, Halliday HL, Juszczak E, et al. Moderate hypothermia to treat perinatal asphyxial encephalopathy. N Engl J Med. 2009;361(14):1349-58.
16. Thoresen M, Whitelaw A. Cardiovascular changes during mild therapeutic hypothermia and rewarming in infants with hypoxic-ischemic encephalopathy. Pediatrics. 2000;106(1 Pt 1):92-9.
17. Levene MI. Management of the asphyxiated full term infant. Arch Dis Child. 1993 May;68(5 Spec No):612-6.
18. Procianoy RS, Corso AL, Schoenardie BO, de Oliveira GPF, Longo MG, Silveira RC. Outcome and Feasibility after Seven Years of Therapeutic Hypothermia in Southern Brazil. Am J Perinatol. 2019.
19. Holmes G, Rowe J, Hafford J, Schmidt R, Testa M, Zimmerman A. Prognostic value of the electroencephalogram in neonatal asphyxia. Electroencephalogr Clin Neurophysiol. 1982;53(1):60-72.
20. Magalhães M, Rodrigues FPM, Chopard MRT, Melo VCdA, Melhado A, Oliveira I, et al. Neuroprotective body hypothermia among newborns with hypoxic ischemic encephalopathy: three-year experience in a tertiary university hospital. A retrospective observational study. Sao Paulo Med J. 2015;133(4):314-9.
21. Variane GF, Cunha LM, Pinto P, Brandao P, Mascaretti RS, Magalhães M, et al. Therapeutic hypothermia in Brazil: a multiprofessional national survey. American Journal of Perinatology. Am J Perinatol. 2019;36(11):1150-6.
22. Procianoy RS. Sociedade Brasileira de Pediatria. [online] Hipotermia terapêutica; 2012. [citado 20 abril 2020] Disponível em: http://www.sbp.com.br/pdfs/hipotermia-terapeutica.pdf
23. Silveira RC, Procianoy RS. Hypothermia therapy for newborns with hypoxic ischemic encephalopathy. J Pediatr (Rio J). 2015;91(6 Suppl A):S78-83.

ANEXO III (Apendix III)

FICHA CLÍNICA (Clinical data)

1. Nome:
2. Registro/Atendimento
3. Data de Nascimento:___/___/___
4. Data do Monitoramento:___/___/___
5. Centro: Estado: SP ( ) RJ ( ) DF/Brasília ( ) RGS ( )
6. UTIN: Hospital ____________

7. Hora/data de início do monitoramento:_____/_____/___________:_____
8. Hora/data de fim do monitoramento: ____/_____/___________:_____
9. HD: principal:____
10. HD: secundários: ____
11. Indicação de monitoramento cerebral: sim ( ) não ( )
12. Laudo da atividade elétrica cerebral emitido: sim ( ) não ( )
6A. Caso sim: Padrão:
Contínuo de voltagem normal ( )
Contínuo de baixa voltagem ( )
Descontínuo ( )
Supressão ( )

Isoelétrico ( )
6B. Caso sim: Ciclo sono-vigília:
Desenvolvido ( )
Imaturo ( )
Ausente ( )
6C. Crise epiléptica:
Ausente ( )
Isolada ( )
Repetitiva ( )
Estado de Mal ( )
6D. Fluxo sanguíneo cerebral:
Dentro de parâmetros normais ( )
Oscilação dentro de parâmetros normais ( )
Dentro de parâmetros alterados ( )
13. Indicação de hipotermia terapêutica: sim ( ) não ( )
14. Realizou hipotermia terapêutica (HT): sim ( ) não ( )
15. Realizou HT: ( ) ativa ( ) passiva
16. Hora/data de início da HT:_____/_____/___________:_____
17. Hora/data de fim da HT:______/_____/___________:_____
18. Efeito adverso HT: sim (   ) não (   ) Qual:______________________________
19. Número total de comunicações remotas: _______
20. Número total de reuniões de discussão de casos: ______
21. Uso de Anticonvulsivantes: Sim ( ) Não ( )   Data início:_ /_ /___<br>Data fim:__ /_ /___Qual(is)?

22. Achado relevante EEG convencional:________________________________
23. Achado relevante de RNM de crânio: _________________________________
24. Achado relevante de USG de crânio: ___________________________________
25. Achado relevante outro exame: _______________________________________
26. Morbidade associada:____
27. Desfecho: alta hospitalar ( ) transferência ( ) óbito ( )
28. Teste de *Bayley* aplicado data:____/___/____
29. Resultado: normal ( ) alterado ( )
